# A community of practice approach to the management of metal resources, metalworking and hoarding in Bronze Age societies

**DOI:** 10.1038/s41598-024-65798-4

**Published:** 2024-07-12

**Authors:** Vana Orfanou, Caroline Bruyère, Andreas G. Karydas, Dragan Jovanović, Filip Franković, Miloš Spasić, Jovan Koledin, Dragan Jacanović, Momir Cerović, Jasmina Davidović, Barry Molloy

**Affiliations:** 1 School of Archaeology, University College Dublin (UCD), Belfield, Dublin 4, Ireland; 2 Laboratory for Artefact Biographies (LAB), University College Dublin (UCD), Belfield, Dublin 4, Ireland; 3 Institute of Prehistory and Early History and Archaeology of the Roman Provinces, Ludwig Maximilian University (LMU) Munich, Schellingstraße 12, 80799 Munich, Germany; 4 Institute of Nuclear and Particle Physics, National Centre for Scientific Research (NCSR) Demokritos, Athens, Greece; 5 City Museum of Vršac, Bulevar Žarka Zrenjanina 20, Vršac, Serbia; 6 Archaeological Museum in Zagreb, Nikola Subić Zrinski Square 19, Zagreb, Croatia; 7 Institute for Prehistory, Protohistory and Near Eastern Archaeology, Heidelberg University, Sandgasse 7, Heidelberg, Germany; 8 Belgrade City Museum, Zmaj Jovina 1, Beograd, Serbia; 9 Museum of Vojvodina, Dunavska 35-37, Novi Sad, Serbia; 10 National Museum of Požarevac, Dr. Voje Dulića 10, Požarevac, Serbia; 11 National Museum of Šabac, Masarikova 13, Šabac, Serbia; 12 Museum of Srem, Vuka Karadžića 3, Sremska Mitrovica, Serbia

**Keywords:** Environmental social sciences, Chemistry, Materials science

## Abstract

The burial of metals in hoards is a trademark phenomenon of prehistoric Europe that may be counterintuitive to perceptions of value nowadays. For the first time here, we establish detailed biographies of a large corpus of hoarded metal objects, providing new insights into how societies in the second millennium BC engaged with their convertible material wealth. We move beyond previous research on prehistoric hoarding commonly focussing on separate questions such as what was placed in hoards, who selected the objects, what were the origins of materials, and where and when they were buried. Analysing *ca.* 200 metal tools and weapons, we use data reduction methods to define technological pathways in the long biographies of hoarded objects extending across the sourcing of materials, production, use, decommissioning, and deposition in the Carpathian Basin. We show how the differential treatment of materials and objects was strongly biased by social decisions across artefact types. We identify shared, standardised signature treatments that crossed over social-spatial boundaries. Our findings bring new insights on the interface between communal and elite wealth management at the intersection of technological reasoning and cultural beliefs in prehistoric communities.

## Introduction

Hoarding of metal objects is a trademark cultural phenomenon of Bronze Age Europe. Distribution and handling of metals in the second millennium BC introduced aspects of economy, including long-distance exchange networks, resource management and standardised systems of value, that are still relatable today^1–4^. Metal hoards took various, often mutually inclusive forms, such as votive (propitiating, soliciting divine forces), placemaking (foundation deposits, markers of events/boundaries) or founders (stock-in-trade, recycling scrap, safekeeping) hoards^5,6^. The often long-term stowing of a valuable commodity with or without the intention of retrieval, bears symbolic and economic attributes less familiar to our attitudes towards value nowadays and can seem ‘irrational and un-economic’^3,5,7,8^. Yet, it was a widespread, long-lived and ‘integral element of the economic system’^3^ of prehistoric Europe.

Hoarding can also be understood as a non-verbal response to times of distress, attempting to mitigate stressors, signalling intolerance to uncertainty^9^. Taking control of uncertainty through material responses, even if seemingly irrational, is inherent to the human condition such as for example the 2020 pandemic-induced panic buying and hoarding of household necessities. By finding comfort in the abundance of resources and/or in the appeasement of the cosmos, hoarding has been an important aspect of the human condition diachronically and a core form of expression of prehistoric communities specifically. Understanding prehistoric hoarding opens a window to the underlying anxieties of pre-monetary communities about an uncertain future and to the management of social stresses and cosmological relationships that were crucial to survival^10^.

Hoards are veritable time capsules preserving traces of activities encompassing many routines of Bronze Age life from ore selection to object making and use, as well as decision making over deposition. They comprised a diverse range of artefact types that had been removed from circulation, ranging from unfinished objects to those heavily worn or broken through use, such as tools, utensils, ornaments, jewellery, weapons, equestrian equipment, and ritual paraphernalia. Furthermore, they disclose important information on the conventions of household, violent, craft, aesthetic, and cultic routines of life. Hoarded objects are commonly broken or rendered unusable (through use or intentionally), but their significance extends beyond their form or function as they are ‘central to transformative processes’^11^. Hoards as assemblages became a meaningful *object,* as well as collections of *things* in the Heideggerian sense. *Objects* may transform to *things* when rendered unusable and lose conceptual links to their original function. Assembled together, these fragments may take on a new identity as a composite *object*—in this case a hoard^12^. These transformations create shifts in meaning^13^ and reflect the *long biographies* of individual objects. We use the term *long biography* to encompass the measurable material traces on a metal object/fragment that may have transitioned through stages of production, use, decommissioning, fragmentation and, finally, hoarding. In the latter stage, the object may be further broken, modified or conceptually stripped of its functional purpose, reverting temporarily or permanently to its intrinsic metal value as a *thing* within a stockpile^14^.

Much of the research on prehistoric metal hoarding has focussed on what went into hoards, why they were deposited, and on issues of fragmentation and geographical distribution in attempts to elucidate cultural meaning(s) of hoarding patterns^7,15–19^. The value of metal is commonly cited, but the nature and materiality of metal in its hoarded state is more rarely discussed. Analytical approaches to hoards tend to focus on select objects in the context of single hoards^20,21^ or specific aspects of the metal, such as its provenance or impurity signatures^22–24^. The divide between contextual and technological studies of hoards reflects a disciplinary gap, which often fails to see hoards as the products of varied long-term technological routines within their social context.

**Figure 1:**
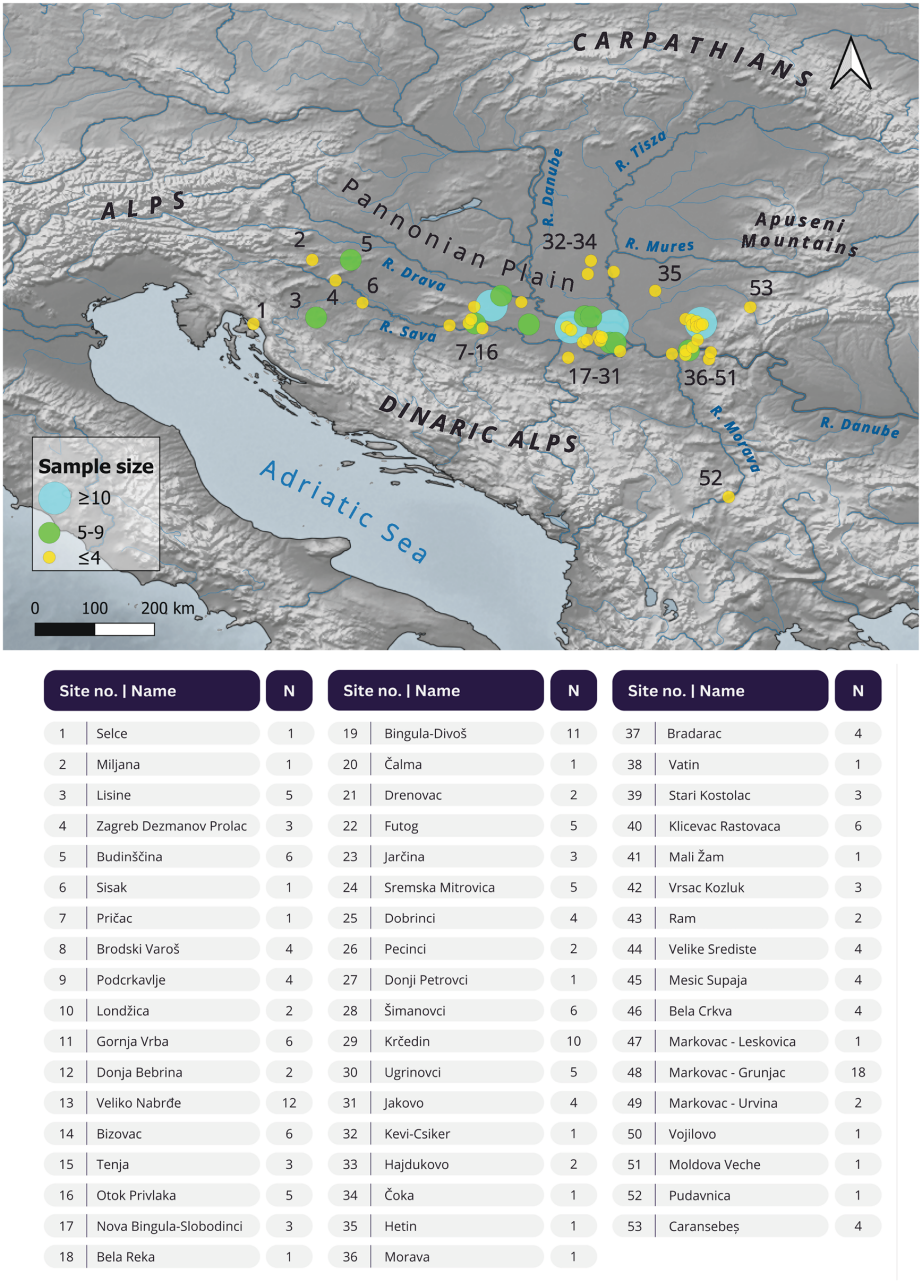
Map showing the location of the sites 1-53 (numbered in increasing longitude values) from which samples are included in the study (N = number of samples) along the southern Pannonian Plain, from the Adriatic coast to the Apuseni mountains (base map from The European Environment Agency; www.eea.europa.eu; detailed coordinates of each site can be found in Suppl. Mat. A1).

Here, we bridge this divide by employing a detailed socio-technological study of 191 Late Bronze Age hoard objects from 53 sites covering a large geographic transect in the south Pannonian Plain from the Adriatic coast to the Apuseni Mountains (Fig. 1). Major rivers, including the Danube, Sava, Drava and Tisza, form significant communication arteries that cut through this region^25^. The diversity of ceramic traditions (the Virovitica, Barice-Gređani, Dubovac-Žuto Brdo and Belegiš II groups), settlement forms and mortuary conventions there indicate variation in cultural expressions and likely political entities. Recent work on the cluster of sites in and around Banat, termed the Lower Pannonian Network, reveals the presence of an important hub of well-organised societies influencing far-flung parts of Europe^26^. Though spatially nebulous, differences in the material expression and enactment of identities may indicate different political units existed moving between east and west in the study area. Similarities, not sameness, nonetheless indicate the presence of a correspondence network within which people and ideas were mobile^27^. In the 13th to 12th centuries BC, crises underlay the collapse of political and economic systems within and beyond the Pannonian Plain including societies of the core areas of the Po Valley and Aegean^28–30^. Gathering and burying of metal hoards increased during these unstable times, with hoarding constituting a likely coping mechanism for those aiming to assert control and manage risk, be it of earthly or celestial origin^5^.

We scrutinise the metallurgical cycle as a culturally specific framework^31–33^ that fused social and technical choices to create routine technological pathways (conventions) in metal production and hoarding that were *translocal* in nature^27^. We employ the concept of *communities of practice* (CoP) ^34^, in relation to metal hoards to assess how signature treatments of raw materials and objects reveal distinct shared behaviours in the later 2nd millennium BC European societies. The CoP concept allows us to identify potentially informal spheres of knowledge that can be distinct from formal cultural units. Functionally speaking, all objects included in this study could in principle have been cast with the same alloy and their blades formed with similar metalworking. Differences (compositional or microstructural) observed in the workflows between different object types reflect choices, which we argue were not random but constitute repeated, culturally informed technological choices. The affordance for choice is significant and may be influenced by numerous factors including the intended purpose/function or appearance of the objects, as well as beliefs or external influences. Taking account of spatial differences in cultural expression and potentially political units across the study area, we evaluate the extent to which metalworking conventions can reveal highly localised and/or translocal systems.

Communities of practice emerged through learning process(es) that linked individual and group agency in craft production that reflect a bottom-up knowledge network^35,36^. Routine choices were expressed as a CoP, namely conceptual planes of knowledge and behaviour whose boundaries may differ from community (political/cultural) boundaries^34,37^. They reflect the beliefs often rooted in inherited traditions^38^, knowhow and skillsets in metalworking^39^ and are embodied by distinct solutions to similar problems adopted by groups and represented in objects; akin to the ‘more than one ways to skin a cat’ saying. These production pathways reflect technological and cultural constraints relating to the natural world (technological reasoning) and the beliefs about that world, respectively^38^. Aesthetic and typological differences occur within our study area (broadly following an east–west bias), affording space for choice within routines. In assuming that choices are constrained by conventions in a manner that is materially evident, a CoP framework allows us to test this hypothesis through scientific methods and define parameters within which choices were made. That, in turn, allows us to explore technology as a vehicle for sustaining translocal aspects of identity evident as practice even when more superficial choices, such as pottery decoration, may serve to express aspects of difference.

## Materials and methods

We examined 191 samples from metal axes, sickles, spears, and swords (Table 1) from LBA hoard contexts in the Pannonian Plain that cover the spread of the aforementioned ceramic traditions (Fig. 1). The objects’ typology dates their making to between 1300 and 1100 BC (Bz D-Ha A1)^40^ and within the broad metallurgical koine of LBA Urnfield bronzes used across much of Europe^41–43^. Even though we can define the spatial distribution of hoards, none of the hoards studied here were recovered through stratigraphic excavation and records on find circumstances are commonly absent or brief^44,45^. The hoard findspots combined with the typology of objects enable our cross-regional comparative perspective. Following Turk’s categorisation of hoards from this wider region^40,46^, the ones studied herein are all categorised as *large hoards of mixed composition*; these usually contain 10’s to low hundreds of objects, fragments of objects, ingots and casting debris. We include a few exceptions such as the single finds of complete swords from the Morava-Danube confluence and Sisak (Fig. 1).

**Table 1:** Sample size of the 4 artefact types (axes, sickles, spears, swords) from the various museum collections.

Museum collection	Axes	Sickles	Spears	Swords	Total
Belgrade	–	–	7	2	**9**
Novi Sad	–	–	6	10	**16**
Pančevo	–	–	–	1	**1**
Požarevac	–	–	10	6	**16**
Šabac	–	–	2	1	**3**
Sremska Mitrovica	–	–	6	16	**22**
Subotica	–	–	1	2	**3**
Vranje	–	–	–	1	**1**
Vienna	–	–	–	1	**1**
Vršac	9	9	11	14	**43**
Zagreb	10	14	23	29	**76**
**Total**	**19**	**23**	**66**	**83**	**191**

**Figure 2:**
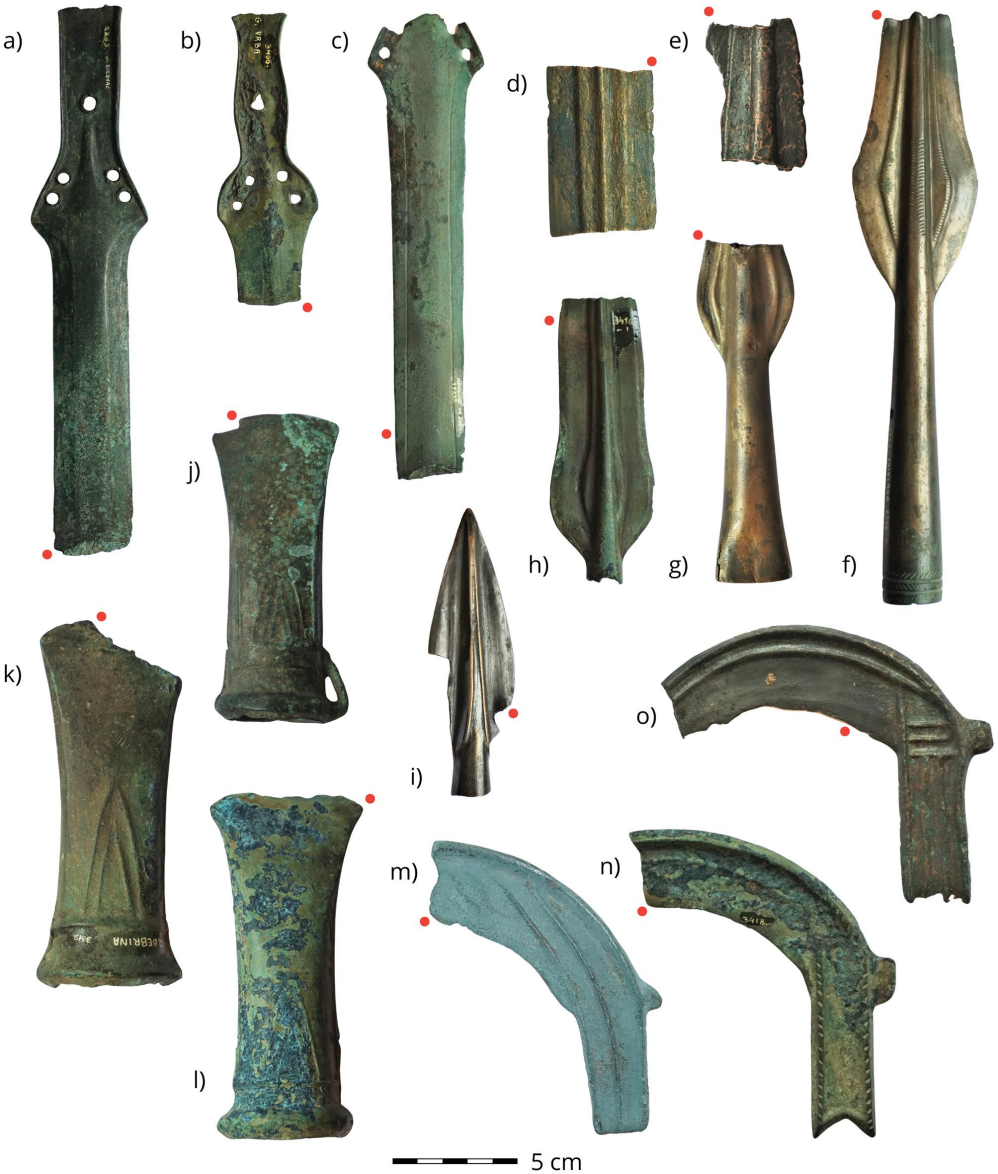
Photographs of representative objects (to scale) sampled during the study (find ID/hoard location): swords (**a**) 2883/Bizovac, (**b**) 3406/Gornja Vrba, (**c**) 17235/Brodski Varoš, (**d**) 17239-2/Brodski Varoš; spears: (**e**) 3945/Bingula-Divoš, (**f**) 2214/Otok Privlaka, (**g**) 10730/Budinščina, (**h**) 3410-1/Gornja Vrba, (**i**) 10081/Pričac; axes: (**j**) 2936/Bizovac, (**k**) 3313 Donja Bebrina, (**l**) 3310/Donja Bebrina; sickles: (**m**) AP1282/Mesic Supaja, (**n**) 3418/Gornja Vrba, (**o**) 11177-38/Brodski Varos. Red dots indicate the sampling point.

With regard to our sampling strategy, we targeted bladed artefacts that enable us to explore the routine pathways in making cutting edges. We focused on swords, spears, sickles and axes because they held inherent potential to be used for varied purposes, including agriculture, craft, butchery, battle, and hunting. With the integrity of objects in mind, samples (< 5 mm in length) were cut from broken objects where practicable (Fig. 2) and prepared using standard metallographic methods.

### Reflected light microscopy (RLM)

Polished surfaces of all samples were examined with a Huvitz HRM 300 optical microscope in reflected light mode at the Laboratory for Artefact Biographies (LAB), at the UCD Centre for Experimental Archaeology and Material Culture at magnifications of 50x-1000x  before and after etching with alcoholic ferric chloride. Observations before etching provide information on the state of preservation and corrosion penetration, metal inclusions and porosity. Etching revealed the nature of the dendritic and granular structures, including shape, size, and characteristics of dendrites and grains, remnant coring segregation, and the positioning of inclusions within the metal matrix. We recorded how metalworking techniques had been applied, what can be described as a sensory *chaîne opératoire*^47^, documenting 37 ordinal and numerical variables to allow for statistical evaluation. We used a metalworking scale to facilitate comparative interpretation of samples and hoards: (1) as-cast, (2) as-cast and cold-worked, (3) partially annealed, (4) fully annealed (with fully grown twins), (5) wrought (a concise label to describe the microstructures that show annealing followed by intense mechanical stress such as hammering), and finally (6) with a fully annealed (FA) or wrought (W) category for the objects that could not be securely placed in either group 4 or 5.

### Micro X-ray fluorescence spectroscopy (micro XRF)

Bulk elemental compositions were established with micro X-ray fluorescence spectroscopy at the Institute of Nuclear & Particle Physics, National Centre for Scientific Research (NCSR) Demokritos, Athens. All analyses took place on sound metal on polished cross-sections following an established calibration protocol for archaeological bronzes^48,49^. Table 2 shows the detection limits for each element analysed (Fe, Co, Ni, Cu, Zn, As, Sn, Sb, and Pb) in wt% and Suppl. Mat. A2 includes the full analysis of certified reference materials (CRMs).

**Table 2:** Table showing the detection limits for each element analysed during micro XRF analysis; all values in wt%.

Element	Wt%	Element	Wt%	Element	Wt%
Fe	0.017	Zn	0.05	Sb	0.3
Co	0.02	As*	0.010–0.075	Pb	0.03
Ni	0.03	Se	0.007	Bi	0.1

*The detection limits for As depend heavily on the Pb concentrations in the same sample and, thus, vary; see Supplementary Table S2 for a detailed account.

### Scanning electron microscopy with attached energy dispersive spectrometer (SEM–EDS)

We conducted semi-quantitative phase identification and additional imaging on selected samples with a Hitachi TM3030Plus scanning electron microscope with an attached energy dispersive spectrometer (operating at 15 kV, a working distance of 8.5 mm, and a deadtime of 20–25%) at the UCD School of Earth Sciences (see Suppl. Mat. A3 for CRM analysis with the SEM–EDS).

### Statistical evaluation and data visualisation

We recorded all collected data (metallographic, elemental, contextual—see Suppl. Mat. A) in numerical and categorical variables (in IBM SPSS Statistics 27), which we used to perform categorical principal component analysis (CATPCA) and correspondence analysis (CA). The former combines quantitative and qualitative data; the latter highlights trends in the sample.

## Results

Our elemental and metallographic data from 191 metal objects shed light on the raw materials, alloying practices, and the methods employed during production and use.

### Bulk elemental compositions

**Figure 3:**
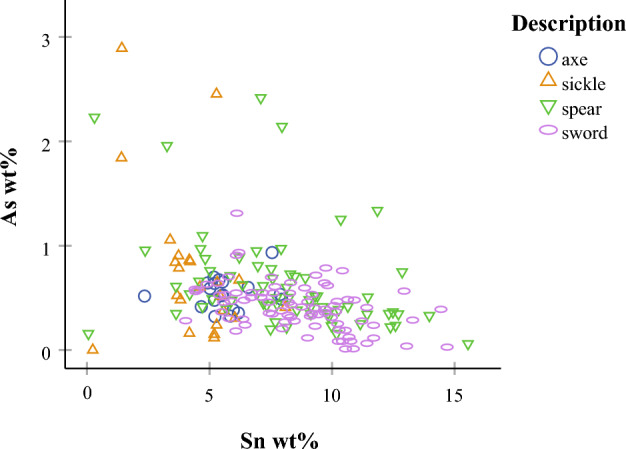
Co-variation scatterplot of As against Sn according to object type (axes, sickles, spears, swords) for the analysed assemblage (n = 191). Spears and swords contain more Sn compared to axes and sickles. Sickles and spears populate the As-rich group (> 1.5 wt% As). A sickle and a sword are the two unalloyed copper objects in the sample.

Here, we interrogate specific alloy patterns and cross reference these with raw materials and technological choices (see Suppl. Mat. A4 and A5 for elemental data). Micro XRF analysis showed that most of the objects (n = 182) comprise a 3–15 wt% Sn bronze (Cu-Sn alloy), with fewer objects of arsenical Cu or bronze with 2–3 wt% As (n = 7) and two objects of unalloyed Cu (< 1 wt% Sn, < 1 wt% As) (Fig. 3). Mean Sn value in the assemblage is 7.5 wt% Sn, but the distribution of Sn shows two peaks at ca. 5 and 8 wt% Sn (see Suppl. Mat. A7 for the Sn histogram). We use this bimodal distribution to categorise bronzes into lower and higher Sn groups with < 7 (n = 76) and > 7 wt% Sn (n = 106) and test these against object types.

Comparison of the bulk compositions and Sn values in the four object types reveals certain discrepancies. The axes and sickles exhibit consistently lower Sn contents, typically in the low-Sn group, with means of 5.3 and 4.3 wt% Sn, respectively. The spears and swords, which populate both Sn groups with higher maximum Sn, contain overall significantly more Sn with means around 8 wt% Sn (Fig. 3). The arsenical Cu/bronze group consists of sickles and spears, while axes and swords contain consistently < 1 wt% As. Finally, one sickle and one spear comprise unalloyed Cu. Overall, spears and swords are similar in their Sn content, but spears and sickles are similar in their As content.

### Minor and trace elements

**Figure 4:**
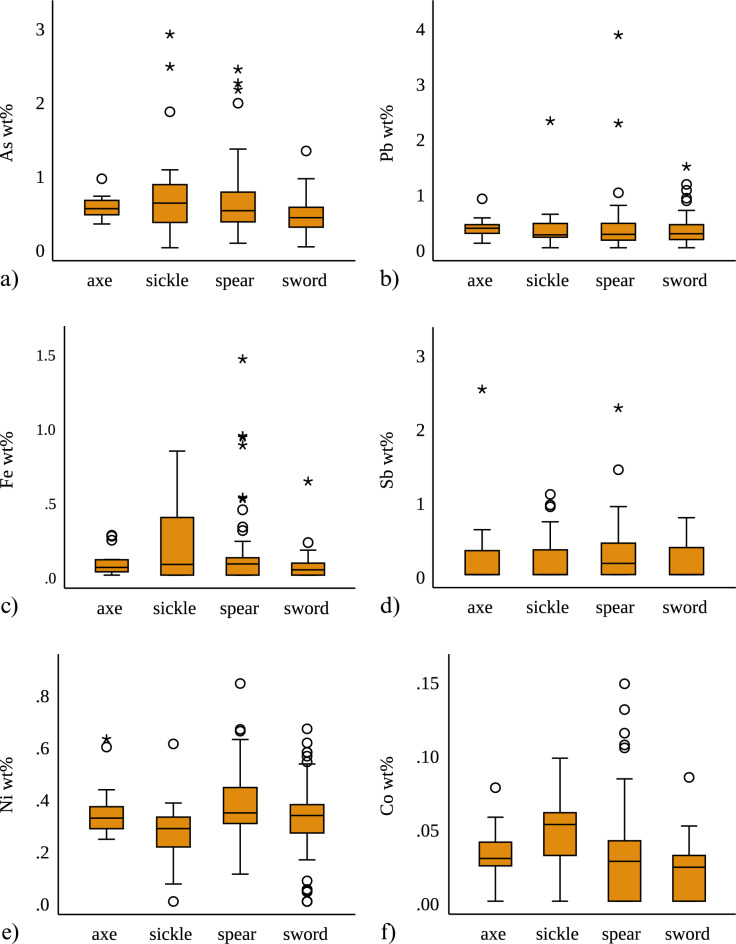
Box plots showing the distribution (min., 1st and 3rd quartile, median, max. values and outliers > 1.5 of IQR; values below LODs have been replaced by zeros) of As, Pb, Fe, Co, Sb, and Ni distribution in the four object types (spears, swords, axes, and sickles) in the sample of 191 analysed objects with micro XRF. Spears and sickles show overall higher As values (**a**) and higher max. values for Pb (**b**), Fe (**c**), and Co (**d**). Sb shows the lowest values in the group of swords (**e**), and Ni shows comparable distributions across the four object types (**f**). Y axis scales reflect the distribution of each element.

Overall, the assemblage shows low trace element concentrations, which are best understood as impurities introduced from the ores (Cu, Sn or Cu/As/Sn), during smelting and/or through re-use and mixing of metals. Table 3 shows the mean, median and maximum values for Fe, Co, Ni, As, Sb, and Pb. As with the bulk compositions, patterns emerge in the trace element concentrations between the four object types (Fig. 4). The pattern noted in the metal impurities follows the observations made based on the As content above. The spears and sickles show similarities with overall higher Pb, Fe and Co contents (Fig. 4b–d), compared to the swords and axes. A similar pattern follows the Sb distribution save for an Sb-rich axe outlier (Fig. 4e). Finally, the Ni content is consistent across artefact types (Fig. 4f). Notably, the swords contain the lowest median values for As, Fe, Co and Pb compared to all other artefact groups.

**Table 3:** Summary table showing the mean, median and maximum values for Fe, Co, Ni, As, Sb, and Pb in the whole analysed assemblage (n = 191).

	Fe	Co	Ni	As	Sb	Pb
Mean	0.11	0.03	0.33	0.55	0.23	0.35
Median	0.04	0.03	0.33	0.48	< LOD	0.26
Max	1.45	0.15	0.84	2.89	2.51	3.84

Values below LODs in the dataset have been replaced by zeros. Se is not included as it was most often undetected with mean and median of 0.001 wt% and max. of 0.01 wt%. < LOD = below limit of detection.

### Metalworking

Of the samples examined (n = 191), 75% (n = 144) showed signs of heat treatment as identified in granular, recrystallised structures (metalworking groups 3–6). By contrast, 25% of the assemblage (n = 47) exhibited as-cast dendritic structures that suggest either no working after casting with generally slow cooling rates or casting followed by hammering/cold-working (metalworking groups 1–2); see Suppl. Mat. A6 for the complete microscopic investigation data and Suppl. Mat. B for representative photomicrographs of all samples.

Metalworking patterns correspond largely to the alloy types. Bronzes are mostly heat-treated (n = 143), and less frequently as-cast or cold-worked (n = 39). Additional discrepancies are noted between the low and high Sn groups. Low-Sn objects tend to be more often as-cast or cold-worked, compared to the high-Sn bronzes, which are more often heat-treated. Unalloyed/arsenical Cu/bronze objects show as-cast or cold-worked structures, while one arsenical object shows signs of only mild heat-treatment (partial recrystallisation).

**Figure 5:**
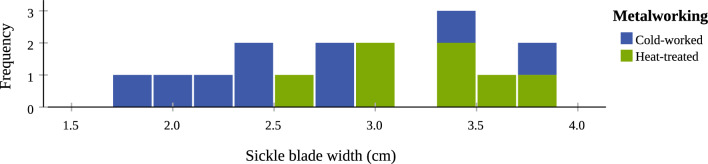
Bar chart showing the metalworking of the sickles according to blade width. Sickles with narrower blades (width ca. < 3.5 cm) are typically—though with a couple of exceptions—cold-worked (as-cast or as-cast and hammered), while sickles with wider blades (width ca. > 3.5 cm wide) are typically heat-treated (wrought or fully annealed).

The four artefact types point to object-specific metallographic structures too (also confirmed by a one-way ANOVA test). The spears and swords share more similarities compared to the axes and sickles, which both show distinct patterns (Table 4). Between *ca.* 75 and 80% of the spears and swords are heat-treated and at least one third of both types is fully annealed, with the rest being as-cast or cold-worked. Of the axes examined, 95% are heat-treated, while *ca.* half of all axes show intense mechanical stress (annealed and wrought). By contrast, the sickles are much more likely to be as-cast and cold-worked. Looking at the width of the sickles’ blades, objects with narrower blades (typically < 3.5 cm) are more often as-cast or cold-worked, compared to sickles with wider blades (> 3.5 cm) which are typically wrought or fully annealed (Fig. 5).

**Table 4:** Summary table showing the percent (%) of the number of objects in each artefact type (axes, sickles, spears, and swords) represented in the each of the metalworking groups (from as-cast to fully annealed).

	As-cast	As-cast and hammered	Partial annealing	Wrought (W)	Fully annealed (FA)	W or FA
Axes (n = 19)	5	0	0	**53**	21	21
Sickles (n = 23)	30*	**30**	9	9	9	13
Spears (n = 65)	23	5	9	14	**36**	15
Swords (n = 84)	7	9	11	15	**38**	18

*Cells ﻿in bold indicate the trends noted in the analysed assemblage.

### Statistical correlations in technological choices

**Figure 6:**
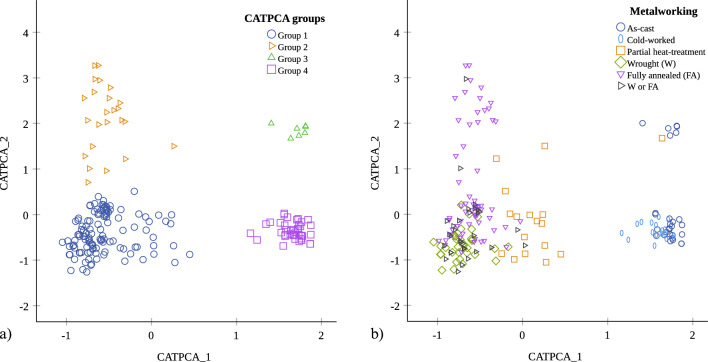
Categorical principal component analysis (CATPCA) in the analysed assemblage of 191 objects based all the metalworking (primary analysis variables) and elemental (supplementary variables) information. We have identified four groups (1–4) based on two CATPCA components (**a**), which largely correspond to metalworking (**b**).

**Figure 7:**
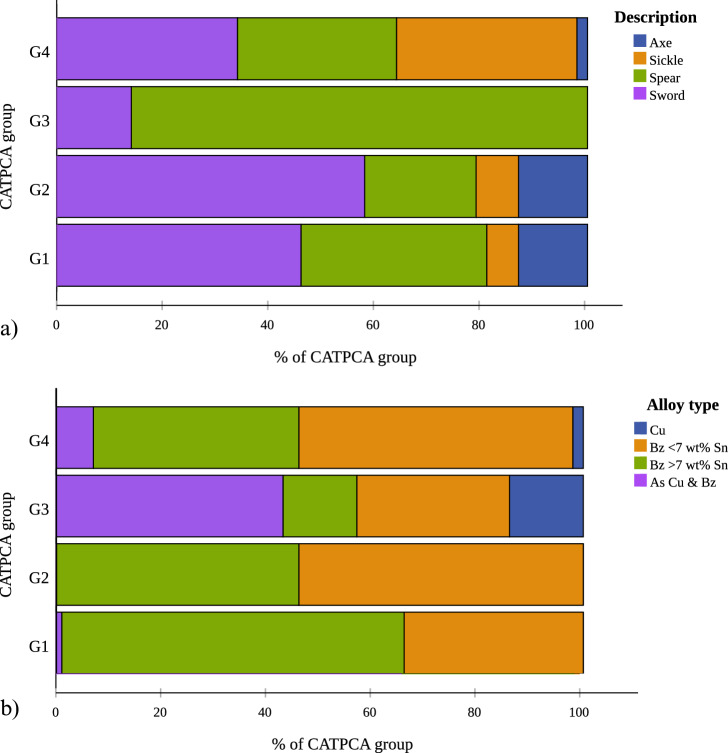
Bar charts showing the distribution of (**a**) artefact types and (**b**) alloy types in % of the Categorical PCA (CATPCA) group size; Bz = bronze. Group 1 of annealed objects (including partially and fully annealed, and wrought microstructures) includes mostly swords and spears, with fewer axes and sickles of bronze (mostly > 7 wt% Sn). Group 2 of fully annealed objects contains mostly swords, with fewer sickles and axes, also of bronze (mostly < 7 wt% Sn). Group 3 comprises as-cast spears and axes with fewer swords and represents a mixture of alloy types, mostly arsenical bronze/Cu and unalloyed Cu, with some low Sn bronzes and fewer high Sn bronzes. Finally, group 4 of as-cast and cold-worked objects comprises mostly sickles, swords and spears with fewer axes of bronze (mostly < 7 wt% Sn) including few arsenical bronze/Cu and unalloyed Cu.

**Figure 8:**
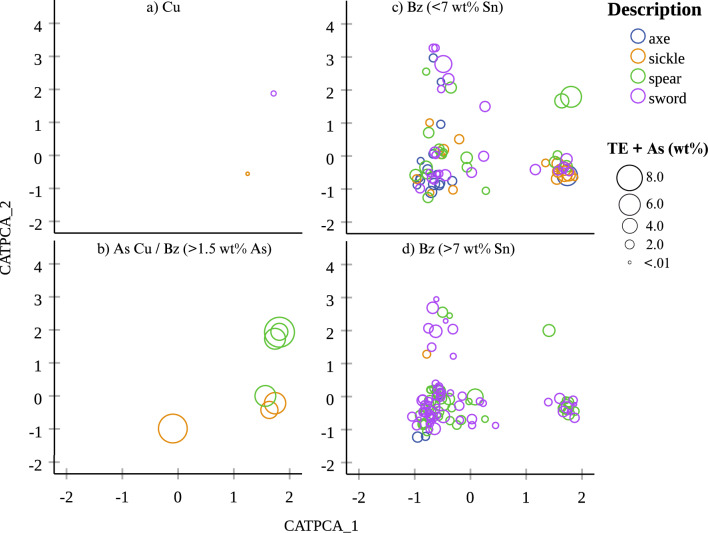
Categorical principal component analysis (CATPCA) in the analysed assemblage of 191 objects showing the distribution of object and alloy types against (**a**—unalloyed copper, **b**—arsenical copper/bronze, **c**—low Sn bronze, **d**—high Sn bronze) the wt% sum of trace elements (Fe, Co, Ni, As*, Sb, Pb) and the CATPCA groups. *As is included in the trace elements (TE) as for most of the samples it is found < 1 wt%. **Bz = bronze.

We sought to bring the elemental and metallographic datasets together for better understanding and illustrating the combined metallographic and elemental patterns by using categorical principal component (CATPCA) analysis with SPSS. For this, we used the metalworking observations as primary analysis variables (Suppl. Mat. A6, columns D to AJ), the elemental data as supplementary variables (Suppl. Mat. A4, columns G to O) and the object description as the labelling variable to create a pair of components. Τhe resulting CATPCA dimensions account for just under 40% of the variance in the sample (see Suppl. Mat. A8 for CATPCA model summary) and upon plotting the components four groups form (Fig. 6a). Despite the partial mediation suggested by the degree of variance, the four CATPCA groups illustrate to a large degree the complete set of our observations based on the combined results. Overlaying the metalworking groups on the CATPCA results, it becomes apparent that CATPCA Groups 1 and 2 cover the heat-treated samples and Groups 3 and 4 the as-cast and cold-worked samples (Fig. 6b). The first CATPCA component (also the most significant) clearly distinguishes between the as-cast and heat-treated samples. The second component represents additional variation based on the elemental composition and artefact type. The CATPCA groups are far from clear-cut as object and alloy types are represented in various ratios within each of the CATPCA groups (Figs. 7 and 8), but notable trends emerge. For example, Group 1 includes mostly high-Sn swords and spears with fewer axes and sickles, while Group 3 is dominated by the As-rich, as-cast and cold-worked spears. Additionally, the lower-Sn as-cast /cold-worked objects in Groups 3 and 4 correlate with higher trace elements (Fig. 8).

**Figure 9:**
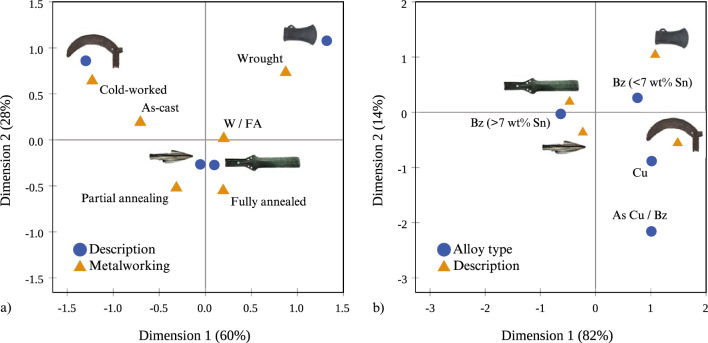
Correspondence analysis (CA) between (**a**) object type (description) and metalworking showing a correlation with a significance (*P* value) of 0.003 explaining a total of 88% of the sample and (**b**) alloy type and object type showing a correlation with a significance (*P* value) of 0.001 explaining a total of 96% of the sample. Both graphs show a strong correlation between how the objects were made and the type of the artefacts in mind.

Correspondence analysis (CA) between the artefact types, metallographic observations and bulk elemental compositions further confirms trending technological choices applicable to most objects in the sample (Fig. 9). Correspondence analysis confirms a strong tendency for the axes to be heat-treated and wrought, and the sickles to be as-cast or cold-worked, whereas the spears and swords better correlate with fully annealed microstructures (Fig. 9a). The latter appear closer to the 0.0 point in the CA plots as they somewhat equally relate to the annealed/wrought groups. Further tendencies are visible between the object and alloy types (Fig. 9b), with trends for low-Sn axes, arsenical sickles, and high-Sn spears and swords.

## Discussion

### Making hoards in LBA Carpathian Basin

Bringing the elemental and metallographic results together (see also Fig. 8), and further building on Tramuž-Orel’s^50^ observations on alloy choices, our detailed look at objects’ long biographies, highlights distinct technological pathways amongst the artefact categories:

- *Axes:* They demonstrate a distinct trend toward heat-treated and wrought microstructures and low Sn content, largely ranging between 3 and 6 wt% Sn (mean 5 wt% Sn), with low trace element concentrations.
- *Sickles:* As with axes, low Sn content is common in the sickles (< 7 wt% Sn, typically 3–6 wt% Sn, mean 4 wt% Sn). However, the higher-than-average trace element concentrations and the occasionally higher As content render them more comparable to the spear group (see below). The tendency for as-cast and cold-worked microstructures is characteristic of this group alone.
- *Spears:* They show the largest Sn range (< 0.1 up to 15 wt% Sn) but generally comprise > 7 wt% Sn bronze (mean 8 wt% Sn) and are occasionally As-rich with higher-than-average trace element concentrations. They resemble the sickles in their patterns of As and trace element contents, but the much larger Sn range, higher mean Sn values, and the more frequent fully annealed microstructures render them more akin to the craft choices for swords.
- *Swords:* They typically contain between 4 and 15 wt% Sn (mean 8.6 wt% Sn) and most often show fully annealed granular microstructures, which match those of the spears. However, their low trace element concentrations are more comparable, but commonly even lower, to the pattern of the axes.

### Sourcing and mixing

Societal demand for metal objects sustained knowledge networks for acquiring and managing metals and making objects^51–53^. Trace element concentrations, including in this case As, are impurities from the Cu source(s); we found no correlation with Sn. Their presence therefore reflects technological choices during sourcing, primary processing (smelting), and potential mixing and recycling^54^. Overall, the analyses of 191 objects indicate that sickles and spears had higher impurity and, occasionally, residual As contents (1–3 wt% As) compared to the low-impurity axes and swords. Even though the Sb levels do not always correlate with As, fahlores from the eastern Alps should be considered as potential contributors to the high As^55–57^. This is further supported by lead isotope analyses of ingots from the same hoards^58^, whereas additional impurities could have been introduced during smelting such as via the fluxes or fuel^59^. Lead isotope analysis for the sickles specifically suggests lack of a single source^58^, which further corroborates our results here.

The axes and more so the swords suggest the presence of more tightly controlled pools of metal by reducing of impurities during remelting operations or by avoiding new metal entering this system^60–62^. The particularly low impurity patterns of the swords could then be explained if a controlled pool of metals/alloys were reserved to be (re)used even as part of remelting/recycling operations. Lead isotope analyses from this region further suggest a system of swords-into-sword recycling and a provenance from Trentino for low impurity swords^58^.

The complex, non-linear sequence of technological events involved several networks of communication and knowledge exchange. This made the high- and low-impurity metals available in secondary workshop locales, indicating distinct technological choices to produce the various blades, directly linked to their specific form and function. This is important for understanding the technological-social interface underlying hoarding. Despite the object-to-thing transition postulated for broken objects upon entering hoards, the functions of objects were still decisive factors during production in this region.

### Alloying and making

The alloying and manufacturing patterns for the four artefact types suggest additional culturally dependent technological choices in blade-making. Alloying and smelting may have taken place in different locations, thus adding a workshop setting into the *chaîne opératoire* of blades. We can divide the assemblage into two Sn groups, namely the axes and sickles with lower mean Sn (4–5 wt%) against the spears and swords with a higher mean (8 wt% Sn). This alloying pattern is different from the metal impurities one (sickles/spears vs. axes/swords). Furthermore, samples with lower Sn are more likely to contain higher As amounts across artefact types, possibly linking technological choices during primary production (sourcing, smelting) with secondary metal treatment (alloying, recycling). The Sn and As content changes the colour^63^ and the physical properties of alloys including their potential for work hardening^64,65^. Lower Sn (3–4 wt%) and higher As for the sickles match data from Slovenia, where axes were found with comparatively low Sn (6–7 wt%), and spears and swords with high Sn^22,50,66^.

Alloying patterns in the sample generally correlate with metalworking traces observed in the microstructures. Objects richer in As and lower in Sn are more likely to be as-cast or cold-worked, compared to objects richer in Sn (and with lower As and overall impurities) which tend to be heat-treated (see Fig. 8). Given that both the Sn content and cold-working tend to increase hardness, the mechanical differences indicated arose from two inter-related technological choices regarding substance (alloy) and form (metalworking).

### Using and hoarding

The function and use of objects, extending to the technology of skill in their making and use, are crucial for understanding past technologies holistically^53,67^. The intended function and actual use of past objects are integral links in the narrative of their biographies. Owing to the large-scale investigation of microstructures, we gained detailed insights into the fingerprinting of use on the metal of each artefact type and revealed that certain objects were heavily used prior to deposition.

The microstructures of the axes and sickles are best explained as results of their use patterns as tools. The group of axes has a strong tendency towards heat-treated and wrought microstructures, which can be the result of repeated hammering as a last step in the metalworking process^68^ or can equally occur due to frequent impacts during use against a hard surface, such as cutting wood. The latter would mask the original metalworking of newly finished axe blades, which was most probably fully annealed. With conservation in mind, samples were cut from incomplete/broken axe-bits (Fig. 2) and ancient breakage may have been a consequence of being used beyond their stability threshold. In any event, this damage pattern corresponds with the microstructures that reveal intense use of axes prior to being removed from use. Similarly but differently, the as-cast and cold-worked structures often observed on sickles (also noticed in LBA Slovenia)^50^ indicate intense use too. Sickle blades may have been work-hardened and heat-treated prior to use, as some sickles preserve annealed microstructures. However, agricultural or household tasks, such as cutting various grasses and crops, would blunt them and it is likely frequent on-the-spot resharpening would keep them serviceable in the field. The broad, thin webs of their blades were well-suited to sustaining a sharp edge despite material loss on each instance. Without skill or resources for annealing in the field or in each household, the worked metal and the original annealed structures of the blade edges were lost, thereby revealing the as-cast core further in, which could have been swiftly hammered. This is further supported by the correlation of the sickles’ width and observed microstructure (Fig. 5), as narrower sickle blades tend to show as-cast and cold-worked structures and wider blades still tend to retain the original heat-treated structures.

The trends observed in the microstructures of blades of spears and swords reveal different use patterns compared to axes and sickles. Spears and swords would not be expected to be hammered against hard surfaces (as with the axes) or used repeatedly and long-term in cutting softer materials (as with the sickles). This difference in use is reflected in and confirmed by microstructures with fully annealed granular structures preserved more often. Metalwork wear analyses has been shown to provide valuable insights into bronze artefact use^69^ and some wear traces are evident on hoarded objects from this region^70^. However, modification related to decommissioning and /or hoarding and the partial representation of once complete objects in hoards creates bias and uncertainty that compromise its value for this present cross-artefact, comparative exercise. Having mentioned the above trends, there are spears and swords in the analysed assemblage that show as-cast and cold-worked microstructures. These may suggest that some mechanically inferior weapons were in circulation (if aesthetics played a larger role) or, more probably in our view, that some fragments analysed were from failed castings that were unfinished before breakage and deposition into stockpiles or hoards (see Fig. 2d).

Breakage patterns^69^ of the hoarded objects reveal additional information about how they were perceived by their social environment. Objects from hoards were commonly broken or rendered unusable leading to the term ‘scrap hoards’^71–73^. Most of the objects we sampled had been broken in prehistory. In our dataset, axes more frequently showed irregularly damaged blades and sickles were most often fractured at various parts of the blades and handles (possibly a result of use), whereas spears and swords were more often evenly broken across the blade (possibly purposeful breakage and/or “killing” of objects) (Fig. 2). This also rendered larger objects into pieces suited for fitting in crucibles for recycling. With only non-refitting fragments of swords in hoards, damage had potentially occurred to *other* non-deposited portions that rendered the object unserviceable^70^. In addition, miscast objects were deposited, as suggested by their macroscopic examination (unfinished objects) and their microscopy (unworked, as-cast microstructures) (Fig. 2d—spear 3945). All the above suggests that the various objects were used differently and deposited at different stages of their use-life.

### Communities of practice in late prehistoric metallurgy and hoarding

**Figure 10:**
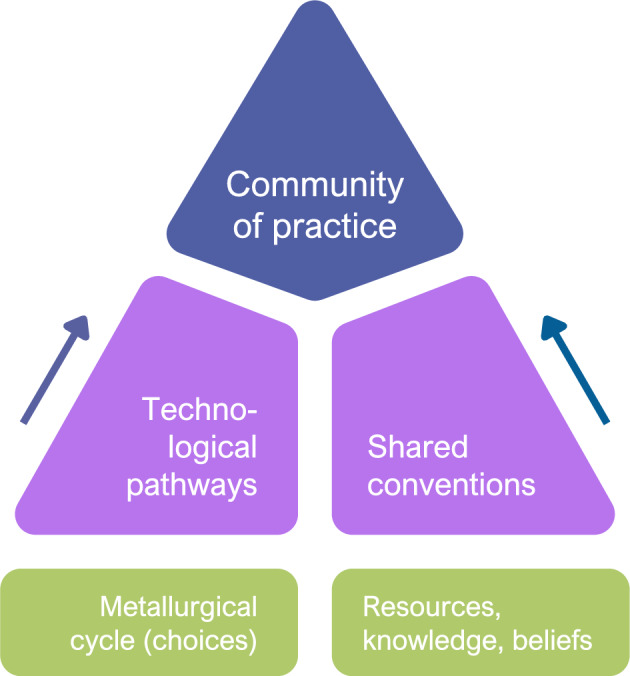
Schematic representation of the formation of communities of practice, which stem from the consolidation of technological pathways and shared conventions in the production of metals based on certain choices along the metallurgical cycle in relation to the available (material) resources, (technological) knowledge and (symbolic) beliefs.

Hoards, rather than end points in object biographies, act as new beginnings of the artefact collections as meaningful assemblages, effectively re-socialising *things* to *objects*. Here, we start from the hoard assemblages to trace the shared socialised materialities of the finished objects back to the raw materials as mirrors of past technological pathways (similar to Kuijper’s sensory *chaîne opératoire*^47^, but extending beyond metalworking to include primary production and use) and worldviews (beliefs) behind prehistoric hoarding of metals. Based on the material restrictions and social perceptions that applied to the metallurgical cycle including the available resources and knowledge, we trace shared conventions in the assemblage that constitute an overarching CoP across a wide geographical transect in the Pannonian Plain (Fig. 10).

There are clear tendencies within each of the artefact types from the choice of raw materials, through to the specific metalworking and use patterns (Figs. 8 and 9). For instance, an axe was made but also treated (used, deposited) differently to sword or a sickle. The nature of the metal, such as seen in the impurity concentrations, and the alloys, as well as the intended functions (utilitarian and symbolic) of the objects were decisive factors that shaped their biographies. Sickles and spears tend to have higher As and overall trace element concentrations compared to axes and swords. Axes and sickles show different alloying, use and often breakage and, thus, deposition patterns compared to spears and swords. Overall, the groups of axes and sickles were made for heavy-duty wear-and-tear, while the spears and swords were used in ways that did not necessarily leave visible traces on their microstructures.

The swords, specifically, stand out and were possibly made from a more strictly controlled pool of metal (as also suggested by lead isotope analyses)^58^ that could suggest quality control^62^. One plausible explanation lies in cultural belief rather than technological reasoning, if producing a good sword required the re-use of metal from proven good swords (metal pooling). This rather distinct technological sword-ecosystem co-existed with the much different technological choices surrounding the social lives of the axes, sickles, and spears that afforded comparatively more space for technological variability. All these artefacts could have potentially been made in the same way, but our results show that often function was a guiding principle in resource, craft and deposition treatment.

**Figure 11:**
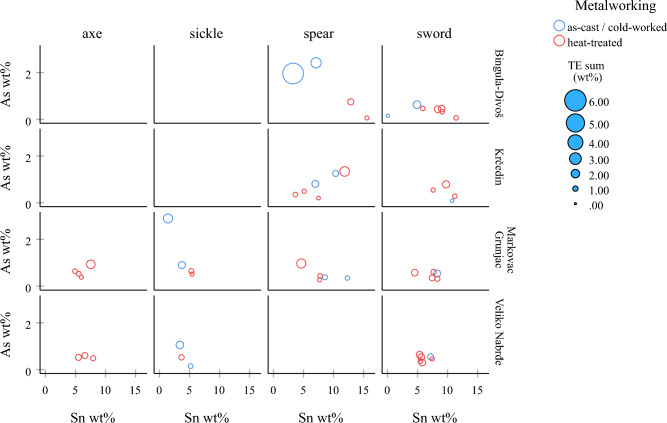
Biplots of As against Sn according to artefact type and metalworking, also indicating the sum of trace elements (TE; scaled and normalised to the Cu content) for the four hoards with > 10 samples in the analysed assemblage (Krčedin, Bingula-Divoš, Veliko Nabrđe, Markovac Grunjac, site numbers 13, 19, 29, and 48 in the map of Fig. 1).

**Figure 12:**
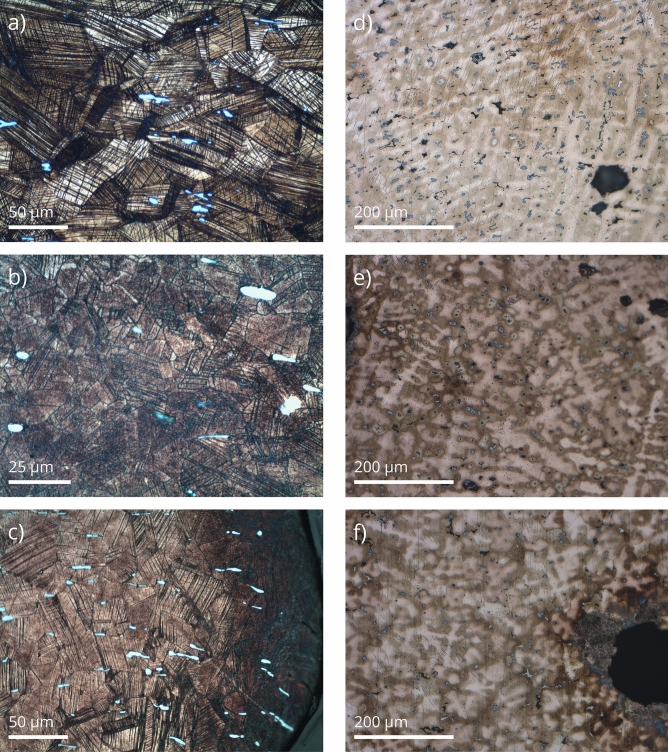
Photomicrographs of axes (**a**—AP10634, 500x, **b**—AP10636, 1000x, **c**—AP10638, 500x,) and swords (**d**—AP10473, **e**—AP10477, **f**—AP10482; all 200x) from Markovac Grunjac that show close to identical wrought (axes) and as-cast (swords) microstructures, which relate to shared communities of practice. Even if these photomicrographs are not identical between them, given the variability expected even within single objects, their similarities are heightened when compared against the backdrop of the full metallographic data set. Field of view: 200x = 650 μm, 500x = 270 μm, 1000x = 130 μm.

The observed shared conventions are present across the hoards examined and are best illustrated in the hoards represented with more than 10 samples (Krčedin, Bingula-Divoš, Veliko Nabrđe, and Markovac Grunjac) (Fig. 11). Given the likely local-scale trade of scrap metal, and the recurrent patterns across hoards, this suggests highly similar attitudes to and technological choices around the long biographies of objects. For example, three axes and three swords from the Markovac Grunjac hoard show metallographic structures that not only are heat-treated with mechanical stress and as-cast respectively, but they are also very close to being identical to each other (Fig. 12). The similarities are such that a single workshop/craftsperson origin could be argued.

These discrepancies within and across the object categories and hoard findspots, are representations of a set of shared conventions by the cultural groups in the southern Pannonian Plain providing a rich metallurgical backdrop for Late Bronze Age hoarding in southeastern Europe. In contextualising these discrepancies, instead of dismissing them as outliers, we can start making sense of the characteristics of a regional trans-local CoP. Even though a hierarchy of technical proficiency has been noted in similar assemblages, for example metalwork from Britain^74^, here we choose not to assign levels of skill or a modern sense of quality/optimisation to the various technological pathways observed between the artefact types. We prefer to understand the latter as the result of shared conventions regarding the material world and appropriate social lives of metal objects. A craftsperson (or a workshop team) could be aware of the technological pathways that were culturally suitable based on an established CoP and still could have made a sickle using one pathway and a sword another (= one CoP dictating differentiated, yet shared, technological pathways). While greater control and potentially time investment surrounded the making of swords to that of the axes, the same smith may have forged both following the shared conventions suited to each according to what they considered as appropriate within the broader context of this regional CoP. Any outliers to the CoP may reflect influence of imports or minority elements within the CoP, whether they be experimental, inexperienced, expedient, incidental or local-specific smithing choices.

The underlying perceptual differences between the four object types were variably reflected across choices in raw material, alloy, crafting, and routine maintenance. These were trends not rules, and they indicate biases in mentalities and beliefs. The discrepancies in production modes reveal differentiated and tiered economy dynamics in the LBA Carpathian Basin. Metallurgical output supplied *consumers* with objects for routine household tasks (e.g., axes and sickles), but also *prosumers* (‘professional’ consumers) with objects that not only looked different macroscopically but were also made in distinct ways and with different resources. Individuals of greater influence, such as individuals with an adopted warrior identity, would be commissioning and using the latter. The fact that the spears show different trace element patterns (tendency for higher impurities) compared to the swords, further strengthens the idea of a *prosumer* market for the swords specifically. This, then, has an avalanche of implications regarding the social and symbolic role of the swords in late prehistoric southeastern Europe.

The existence of a spatially extensive metallurgical CoP in the southern Pannonian Plain further highlights facets of the organisational depth of late prehistoric European societies. Childe’s model of travelling smiths or newer models of travelling apprentices provide a human face to the networks through which this CoP was maintained^75–77^. Furholt’s^27^ non-correspondence system, in which some individuals within a residence group may hold deep (or deeper) social relations with others outside that, is also pertinent to understanding how our results reflect past stability/mobility patterns and knowledge exchange networks. Finally, as metalworking requires apprenticeship, training may have been a routine means through which trans-local relations were sustained, different to but not fully unlike the model of fostering in prehistoric societies^78^.

## Conclusions

Late Bronze Age metal hoards in the Carpathian Basin are more than the sum of their parts. They are stand-alone entities that constitute varied microcosms of past engagements between people and objects, from the makers through to the users and depositors. In this study, we have examined in detail ca. 200 metal objects to build a collective narrative about the social world surrounding prehistoric hoards. This assemblage approach to hoards allows us to reflect upon past perceptions about shifting intersections between people, *things* and *objects*, thereby bridging the natural and social environments. Our investigation revealed entangled and routine, socially embedded biographies of bladed metal objects from ore sourcing through to making, using, and depositing. These long biographies point to distinguishable, but tiered and shared conventions forming a community of practice (CoP). This comparative technological approach to objects with broadly shared material properties (copper alloys), morphological features (blades) and a certain destiny (hoarding) revealed biases in resource management and the social lives of metal objects, which were shared by the cultural groups of the southern Pannonian Plain.

Elemental, microscopic, and statistical analyses reveal the specific ways in which the axes, sickles, spears, and swords were created, used and/or deposited. We note looser control of raw materials during sourcing and possible higher rates of recycling in sickles and spears, as opposed to swords and axes. Spears and swords, albeit both classified as weapons (for their primary use, but with more uses possible), each represent distinct conventions within an overarching CoP. We underline a distinct technological *eco-system* for the swords with indications for metal pooling marked by low metal impurities. The latter helps us understand the influence of the original function of the objects on metal recycling. The breakage or intentional damage of metal objects in preparation for reuse (remelting or hoarding) of the metal is commonly perceived as removing the identity from the objects. However, the special treatment of the sword-metal suggests that the object form and function reemerged as decisive factors in resource management.

The forging of bladed objects initially, and their upkeep and repair over time, differentiate further the managing of blade-making that was in part responsive to the natural, but also the social realms. At the same time, aesthetic preferences played an important role as weapons (spears, swords) tend to contain overall higher Sn, compared to tools (axes, sickles). Finally, approaching the end of their long biographies, additional discrepancies occur as each artefact group appears to have been removed from circulation and hoarded at different stages of use. Axes and sickles are deposited more often with strong traces of physical use and fractures from use, compared to spears and swords, which tend to be more often intentionally broken in preparation for hoarding.

Entanglements between people and objects were regulated but not restricted, as evidenced by the varying treatments of different object types as they moved through different biographical states. Yet, at the same time, strong biases according to object types created patterns that signal repeated, even institutionalised, preferences founded upon technological beliefs. Our data show that metal management was controlled, and we would also argue that it was partly standardised within the dominant CoP spanning the southern Pannonian Plain. A dominant CoP identified via a functional object biographical approach, further reveals the strong impact of the worldviews and beliefs around metal objects on the latter’s materiality and embodied tactility.

Finally, we have shown how participation operated across a wide and culturally diverse area through the lens of the macroculture of metalwork.  It extended across traders/distributors, smiths and their apprentices, end-users and ritual or economic agents managing hoards in Bronze Age Europe. This goes beyond technological determinism, as distinct (path)ways are represented within and across artefact types, as well as hoarded things. Understanding shared conventions under a community of practice in hoarded assemblages revealed the intricate and inter-woven links between technology, materials, and culturally formed approaches to metal objects in LBA societies.

## Supplementary Information

Supplementary Tables.

Supplementary Information.

## Data Availability

All data generated during this study are included in the publication as supplementary materials.
