# Author Correction: A community of practice approach to the management of metal resources, metalworking and hoarding in Bronze Age societies

**DOI:** 10.1038/s41598-024-78806-4

**Published:** 2024-11-19

**Authors:** Vana Orfanou, Caroline Bruyère, Andreas G. Karydas, Dragan Jovanović, Filip Franković, Miloš Spasić, Jovan Koledin, Dragan Jacanović, Momir Cerović, Jasmina Davidović, Barry Molloy

**Affiliations:** 1 School of Archaeology, University College Dublin (UCD), Belfield, Dublin 4, Ireland; 2 Laboratory for Artefact Biographies (LAB), University College Dublin (UCD), Belfield, Dublin 4, Ireland; 3 Institute of Prehistory and Early History and Archaeology of the Roman Provinces, Ludwig Maximilian University (LMU) Munich, Schellingstraße 12, 80799 Munich, Germany; 4 Institute of Nuclear and Particle Physics, National Centre for Scientific Research (NCSR) Demokritos, Athens, Greece; 5 City Museum of Vršac, Bulevar Žarka Zrenjanina 20, Vršac, Serbia; 6 Archaeological Museum in Zagreb, Nikola Subić Zrinski Square 19, Zagreb, Croatia; 7 Institute for Prehistory, Protohistory and Near Eastern Archaeology, Heidelberg University, Sandgasse 7, Heidelberg, Germany; 8 Belgrade City Museum, Zmaj Jovina 1, Beograd, Serbia; 9 Museum of Vojvodina, Dunavska 35-37, Novi Sad, Serbia; 10 National Museum of Požarevac, Dr. Voje Dulića 10, Požarevac, Serbia; 11 National Museum of Šabac, Masarikova 13, Šabac, Serbia; 12 Museum of Srem, Vuka Karadžića 3, Sremska Mitrovica, Serbia

Correction to: *Scientific Reports* 10.1038/s41598-024-65798-4, published online 12 July 2024

The Acknowledgments section in the original version of this Article was incomplete.

“The authors would like to thank all colleagues who have made this work possible with fieldwork, documentation, and conservation of artefacts. We thank specifically Sanjin Mihelić, Jacqueline Balen and Damir Doračić at the Archaeological Museum in Zagreb, Neda Dimovski from the City Museum of Subotica and Goran Mitrović from the National Museum of Vranje for providing important access to study materials. Funding for Vana Orfanou’s postdoctoral position and analyses for this study were covered by the ERC CoG awarded to Barry Molloy (GA #772753), *The Fall of 1200 BC: The role of migration and conflict in social crises at end of the Bronze Age in South-eastern Europe*. Barry Molloy also thanks Roger Doonan, PI during MSCA Postdoctoral Fellowship (GA #275958), *Metals, weapons and social change around the Adriatic and Ionian seas 2000-1000 BC: A longue durée vista on the impact of military praxis on technology, politics and communication networks*, when the first sampling campaign took place and Peter Northover for advice during the preliminary evaluation of samples. Vana Orfanou would also like to acknowledge her MSCA Postdoctoral Fellowship, *Changing World* (#101067234), at LMU Munich, which funded her position during the final stages of publication of this study.”

now reads:

“The authors would like to thank all colleagues who have made this work possible with fieldwork, documentation, and conservation of artefacts. We thank specifically Sanjin Mihelić, Jacqueline Balen and Damir Doračić at the Archaeological Museum in Zagreb, Neda Dimovski from the City Museum of Subotica and Goran Mitrović from the National Museum of Vranje for providing important access to study materials. Funding for Vana Orfanou’s postdoctoral position and analyses for this study were covered by the ERC CoG awarded to Barry Molloy (GA #772753), The Fall of 1200 BC: The role of migration and conflict in social crises at end of the Bronze Age in South-eastern Europe. Barry Molloy also thanks Roger Doonan, PI during MSCA Postdoctoral Fellowship (GA #275958), Metals, weapons and social change around the Adriatic and Ionian seas 2000-1000 BC: A longue durée vista on the impact of military praxis on technology, politics and communication networks, when the first sampling campaign took place and Peter Northover for advice during the preliminary evaluation of samples. Vana Orfanou would also like to acknowledge her MSCA Postdoctoral Fellowship, Changing World (#101067234), at LMU Munich, which funded her position during the final stages of publication of this study. The publication of this article was supported by the Onassis Foundation Scholars’ Association."

The original Article has been corrected.

