# Supplementary material for: A community of practice approach to the management of metal resources, metalworking and hoarding in Bronze Age societies
Source: Sci Rep. 2024 Jul 12;14:16153. doi: 10.1038/s41598-024-65798-4 (PMC11245533; doi:10.1038/s41598-024-65798-4)
Supplement: Supplementary file 2 — Supplementary Information. [file 41598_2024_65798_MOESM2_ESM.pdf]

Supplementary Material B. Catalogue of photomicrographs

Orfanou et al. Beyond technological reasoning: Identifying how communities of practice shaped the management of metal resources in Bronze Age societies

Table of representative photomicrographs of etched microstructures (with alcoholic ferric chloride), unless otherwise stated, produced during the metallographic observation of the sample included in the study. Total 192 photomicrographs from 191 (2 images included for spear P908). Field of views: 1.3 mm at 100x, 650 μm at 200x, 270 μm at 500x, 130 μm at 1000x.

Find ID & Description	Photomicrograph	Magnification & notes	Find ID & Description	Photomicrograph	Magnification & notes
10.108 sword	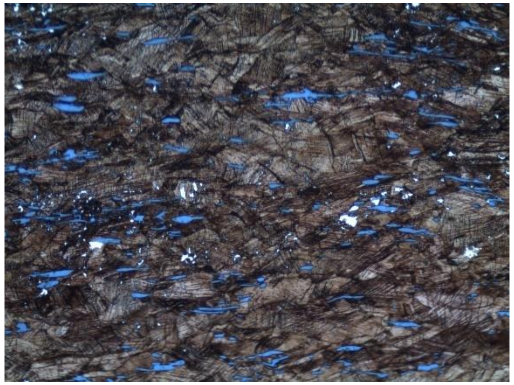	500x	1001 sword	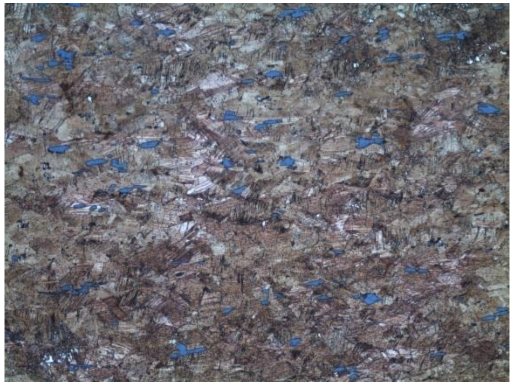	500x
10081 spear	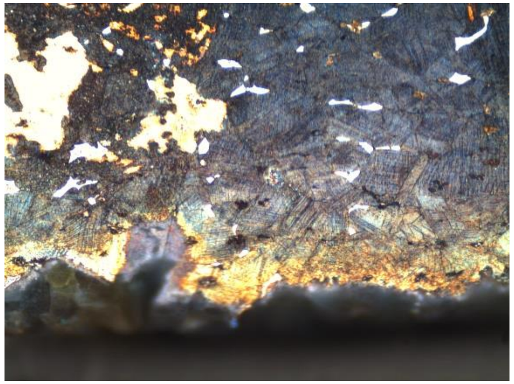	500x	10120 sickle	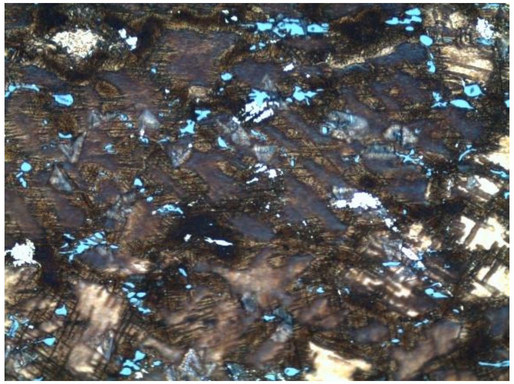	500x

Find ID & Description	Photomicrograph	Magnification & notes	Find ID & Description	Photomicrograph	Magnification & notes
10125<br>sickle	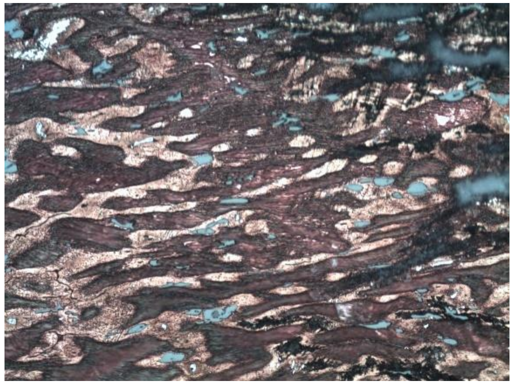	500x	10128-1<br>sickle	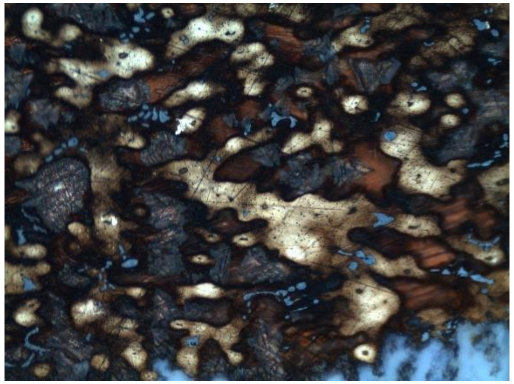	500x
10131<br>spear	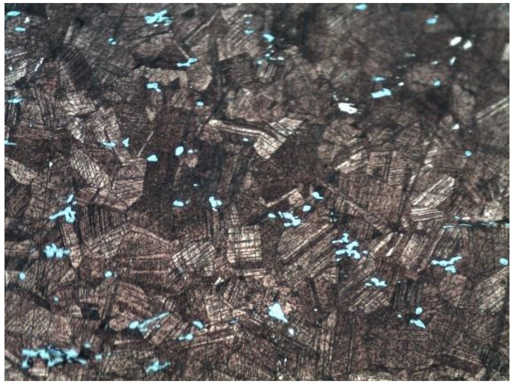	500x	10132<br>spear	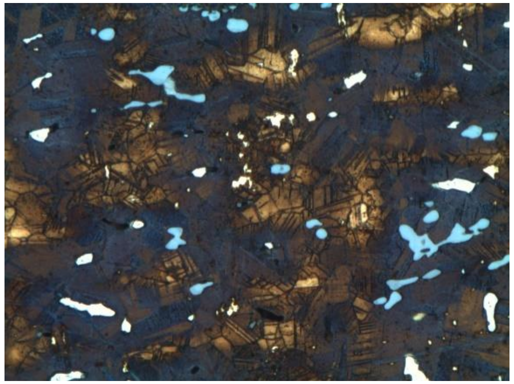	1000x
10157<br>axe	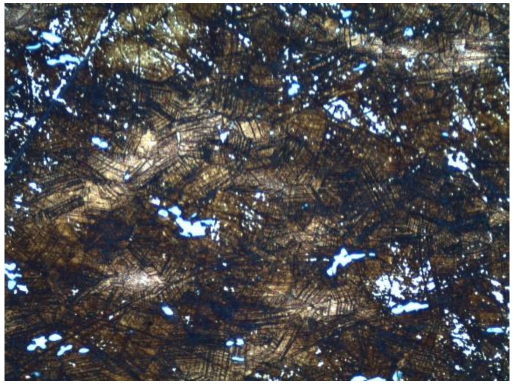	500x	10160<br>axe	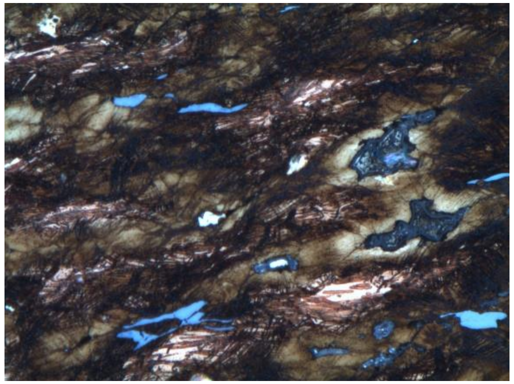	1000x

Find ID & Description	Photomicrograph	Magnification & notes	Find ID & Description	Photomicrograph	Magnification & notes
10162<br>axe	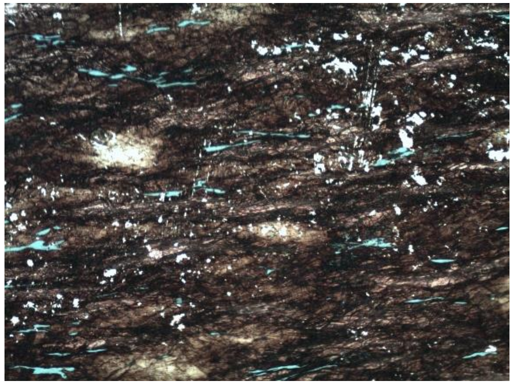	500x	10166<br>sickle	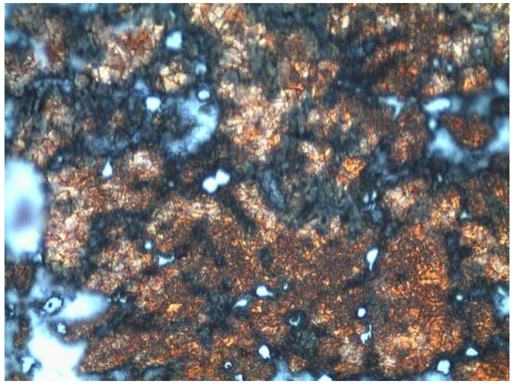	500x
10168<br>sickle	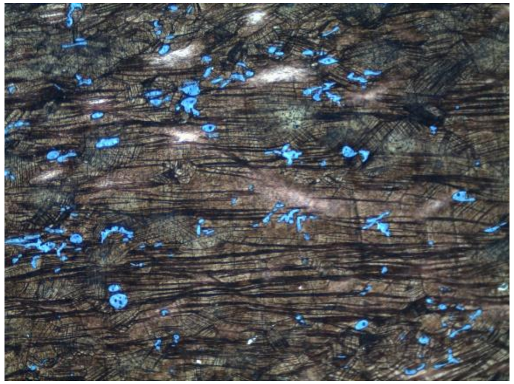	500x	10170<br>sickle	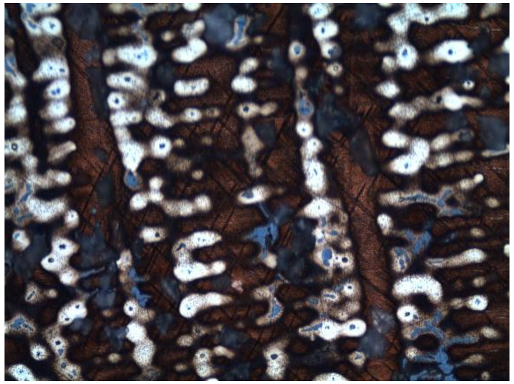	500x
10179<br>sword	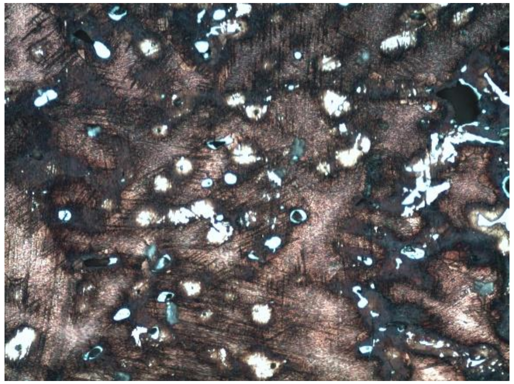	500x	10180<br>sword	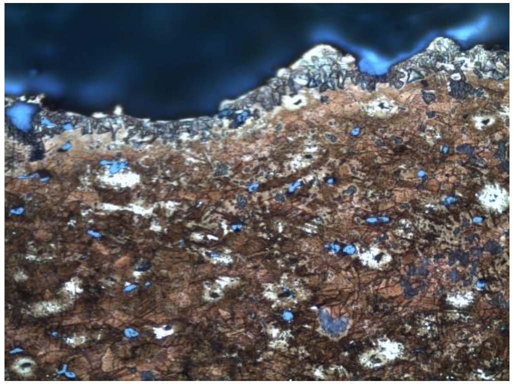	500x

10181<br>sword	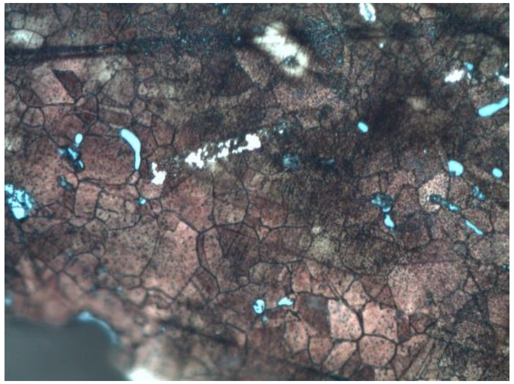	500x	10182<br>sword	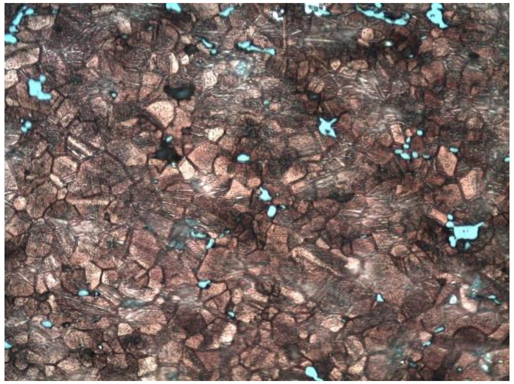	500x
10187<br>sword	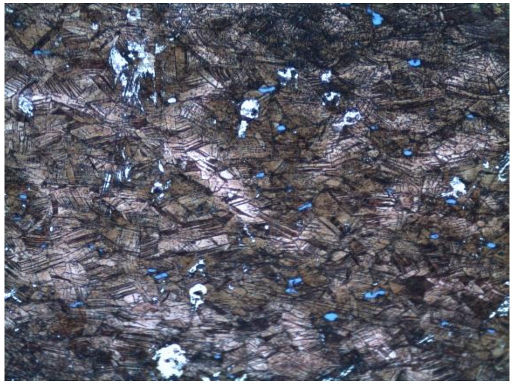	500x	10188<br>sword	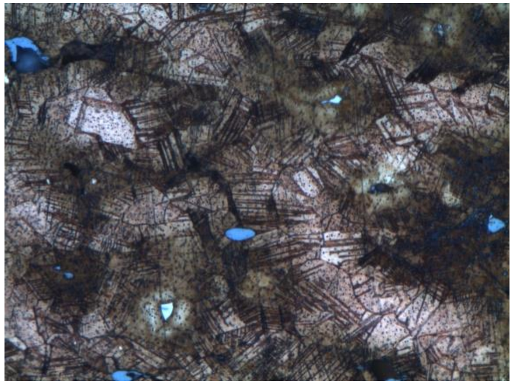	1000x
10458<br>sword	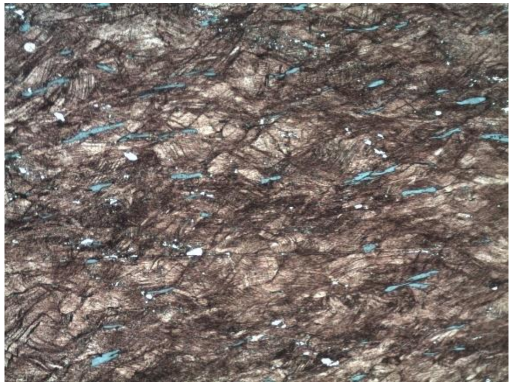	500x	10730<br>spear	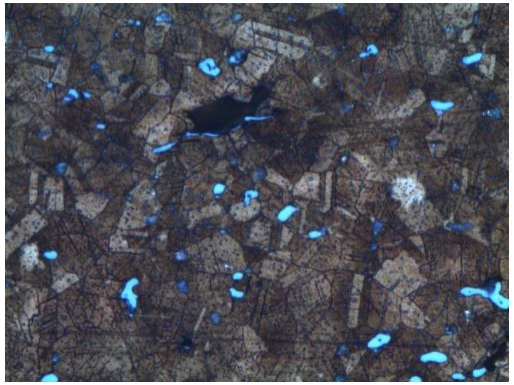	1000x

10732 spear	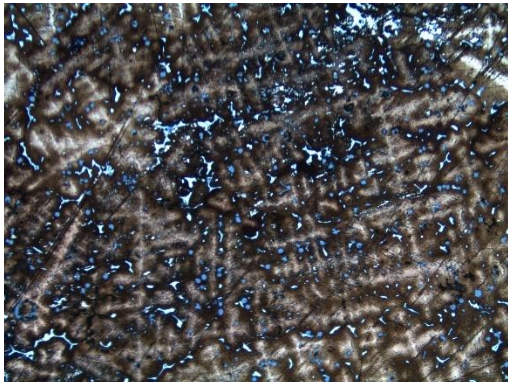	200x	10774 sword	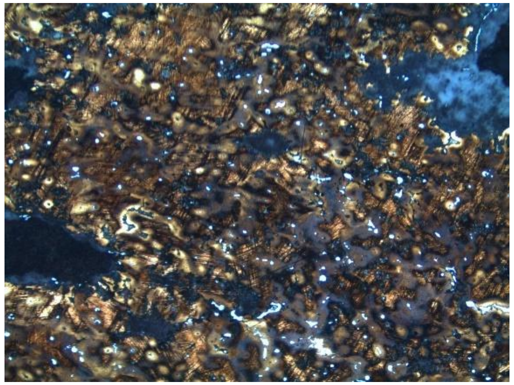	200x
10776 sword	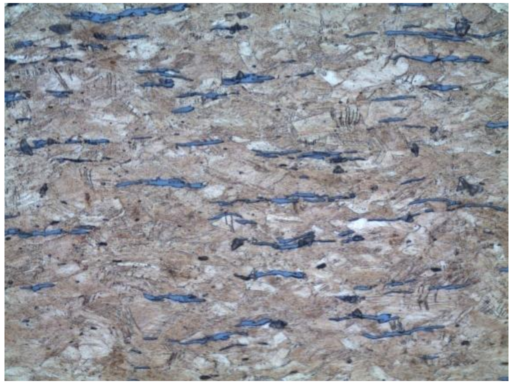	500x	10777 sword	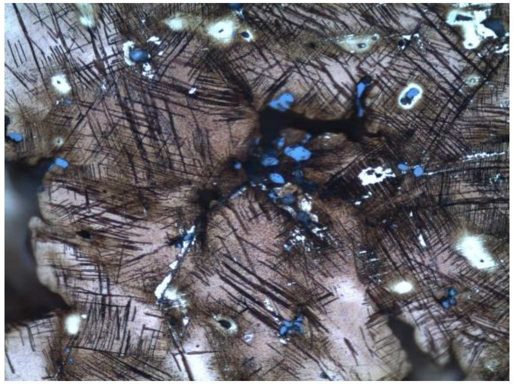	500x
10782-1 spear	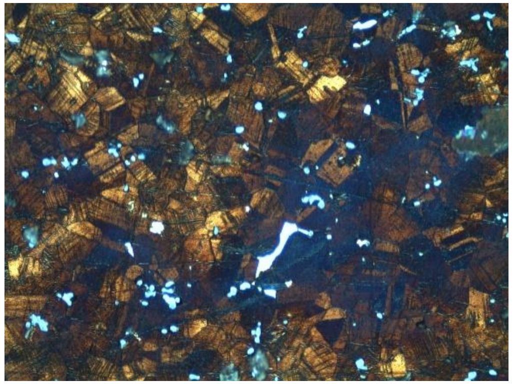	500x	11177-38 sickle	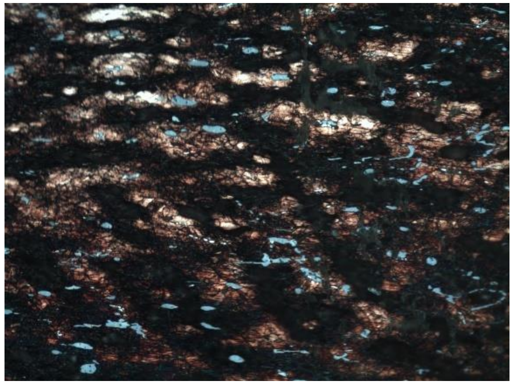	500x

1179<br>axe	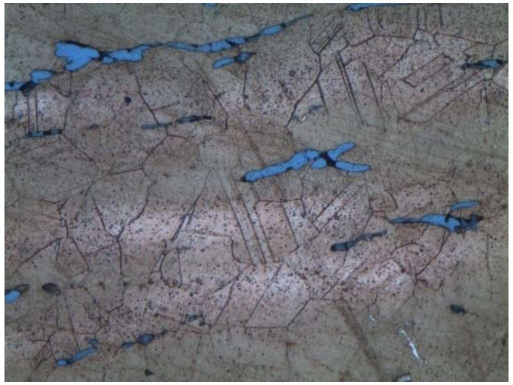	1000x	1181<br>axe	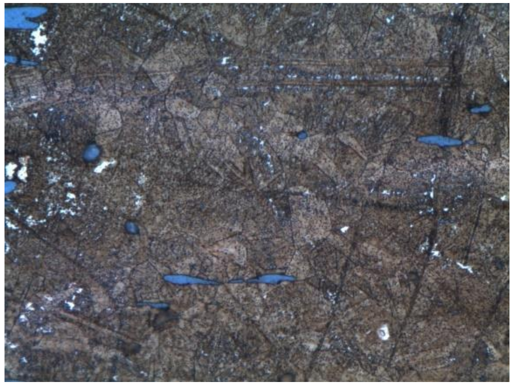	1000x
1194<br>sword	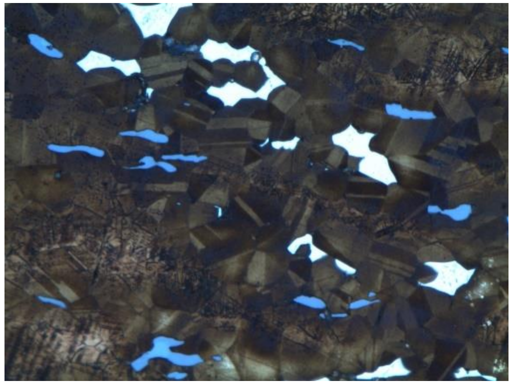	1000x	1205<br>sword	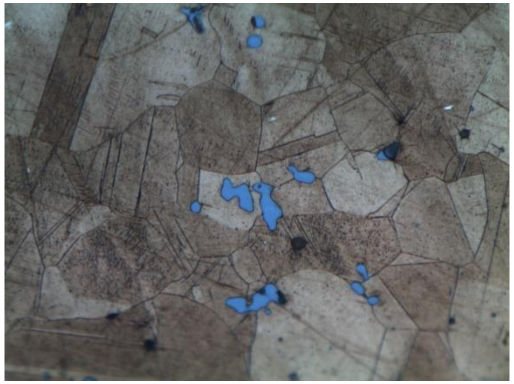	1000x
1529<br>spear	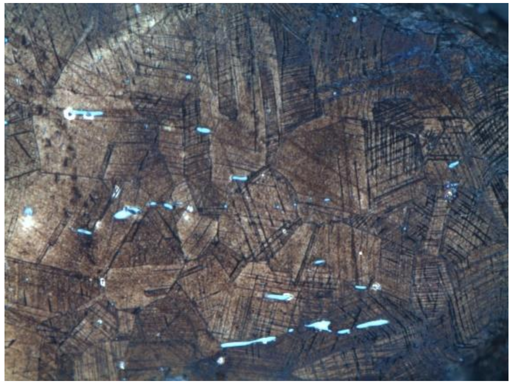	1000x	1532<br>spear	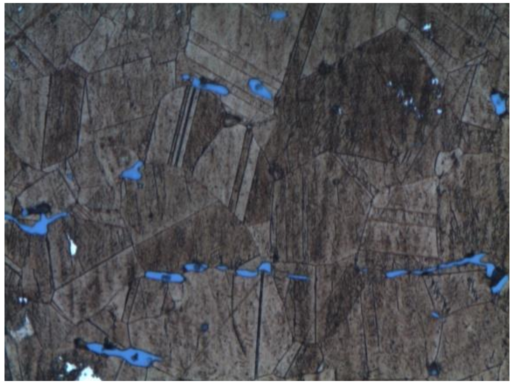	1000x

1533<br>spear	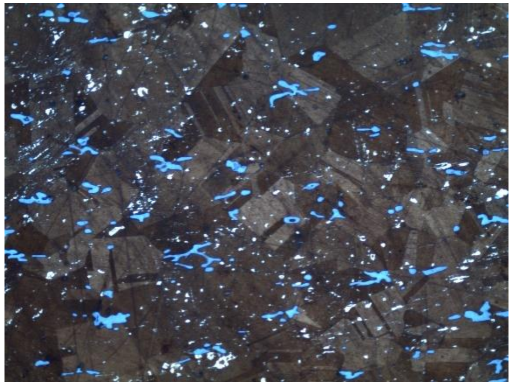	500x	1547/1<br>axe	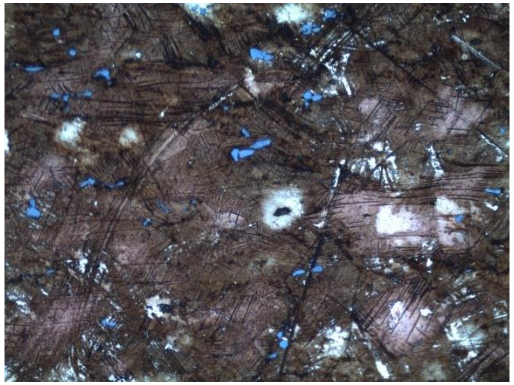	500x
1547/5<br>sickle	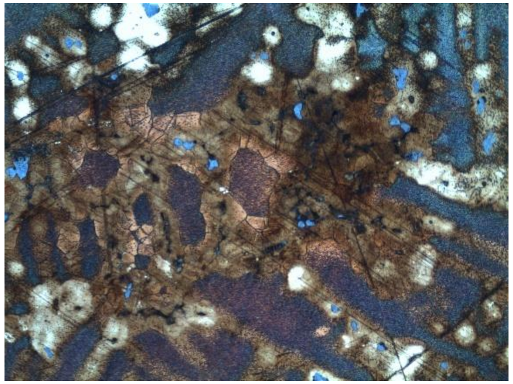	500x	17235<br>sword	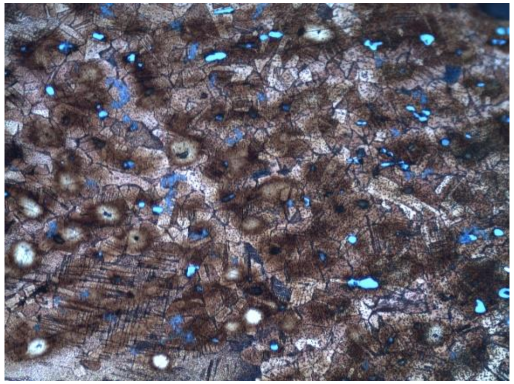	500x
17239-2<br>sword	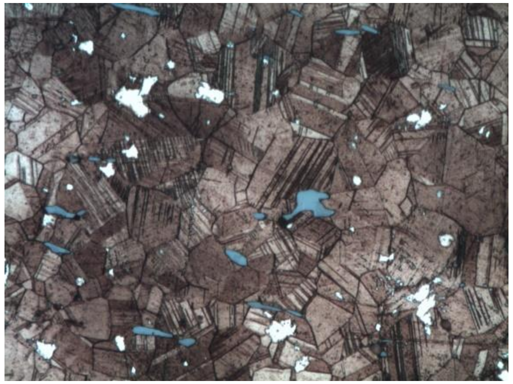	500x	18.3400<br>sword	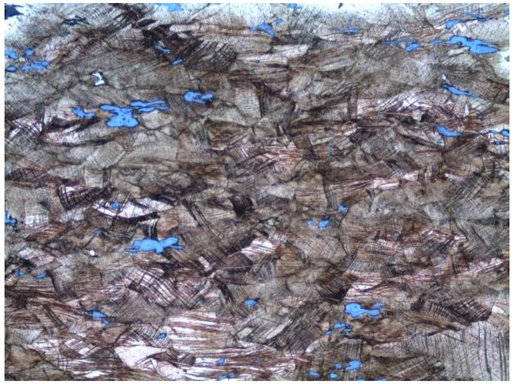	500x

1989.14<br>sword	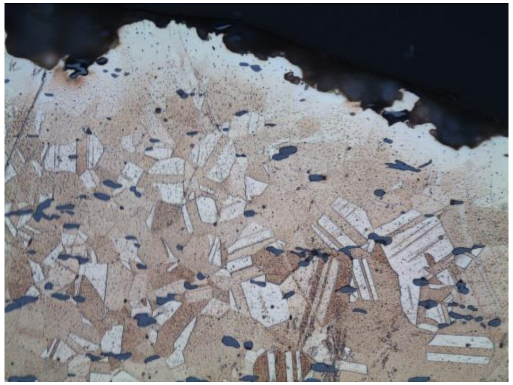	500x	204<br>spear	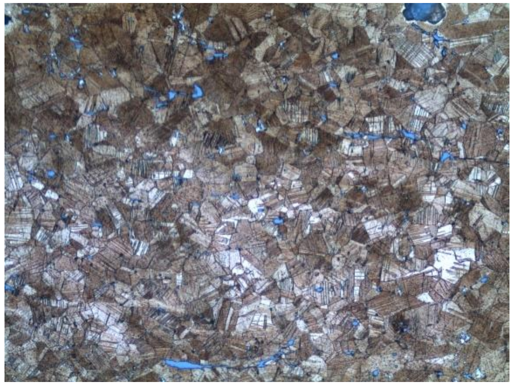	500x
2103<br>sword	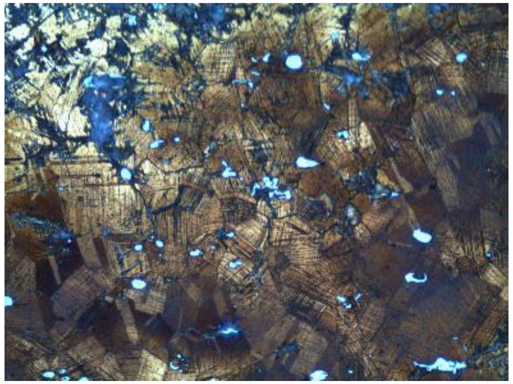	500x	2104<br>sword	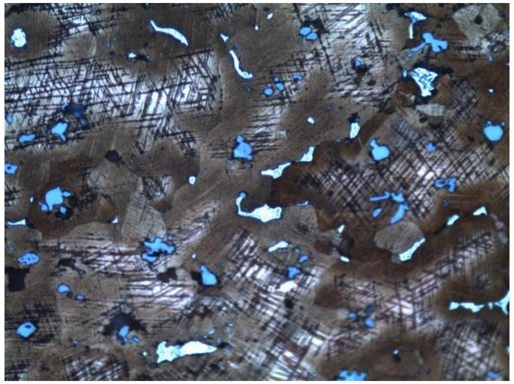	500x
2105<br>sword	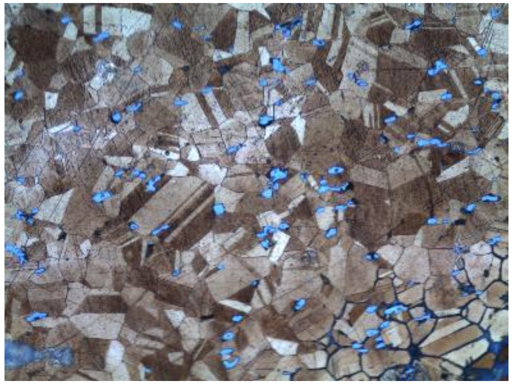	500x	2130<br>sword	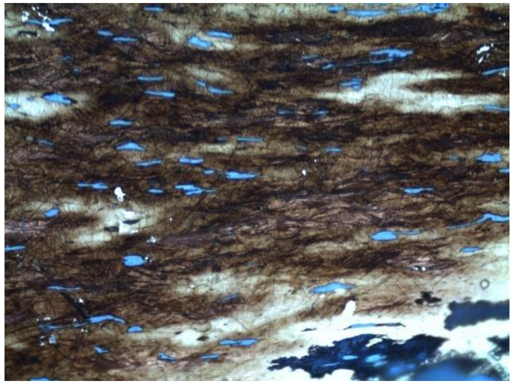	500x

2131<br>sword	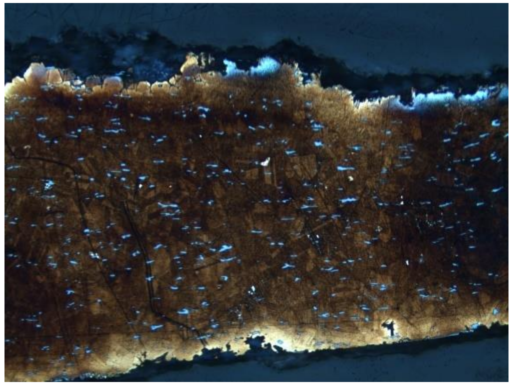	200x	2132<br>sword	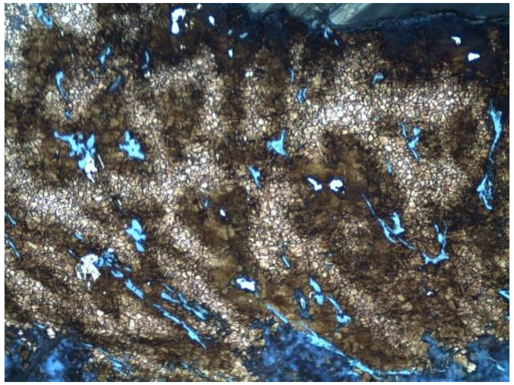	500x
21334-2<br>sword	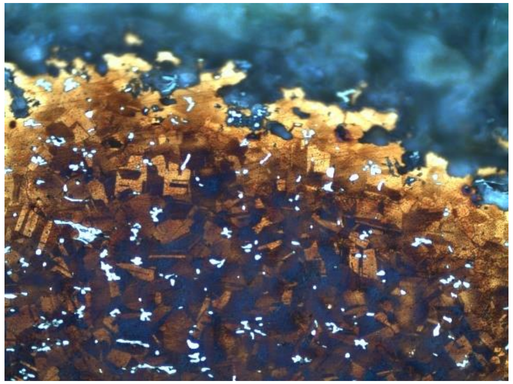	500x	2134<br>spear	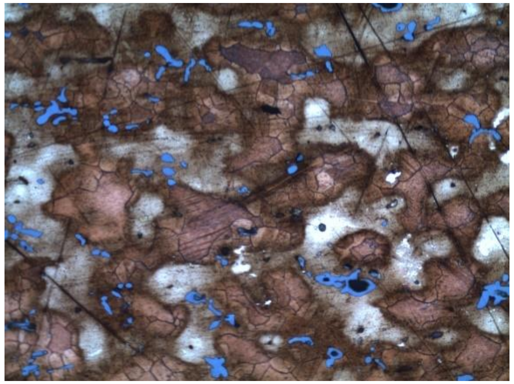	500x
2137<br>spear	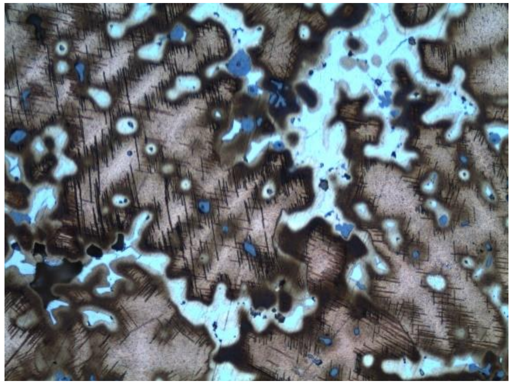	500x	2138a<br>spear	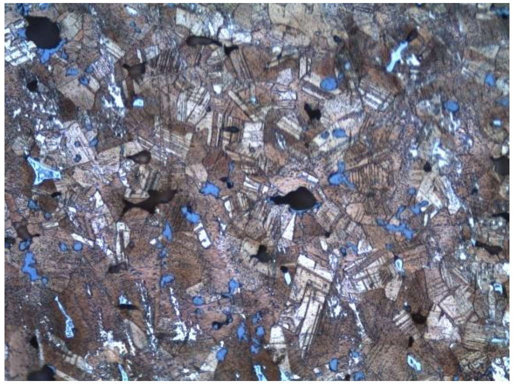	500x

2138b<br>sword	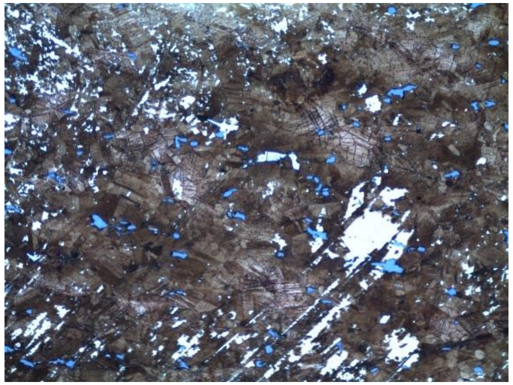	500x	2165<br>sword	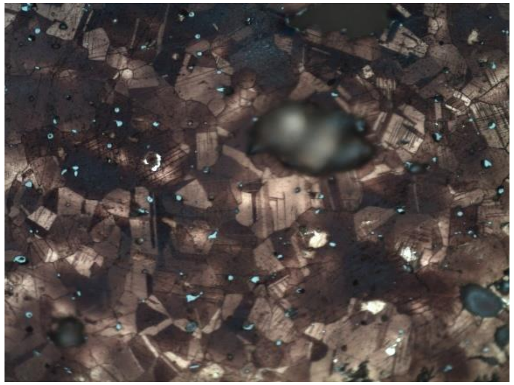	500x
2166<br>sword	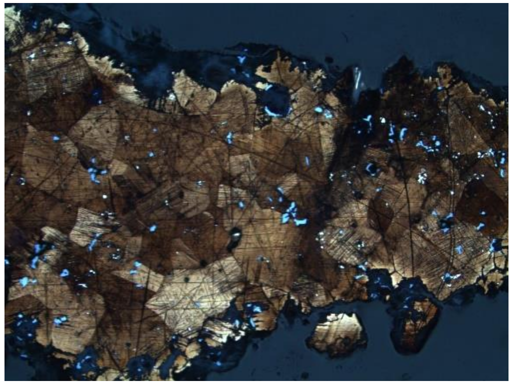	100x	2181<br>sickle	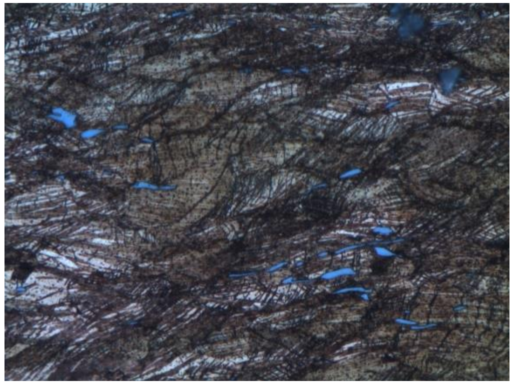	1000x
2214<br>spear	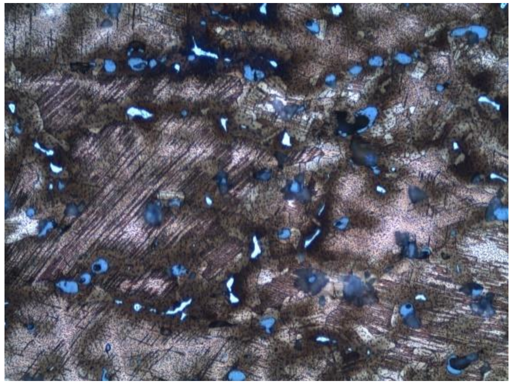	500x	2216<br>spear	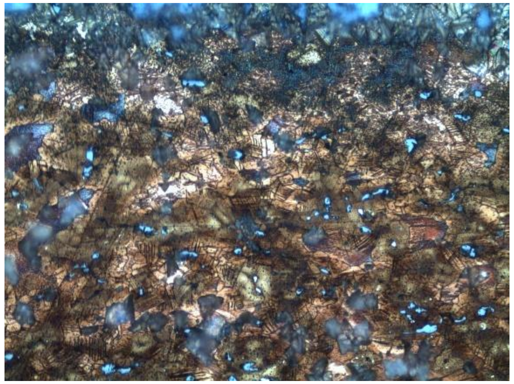	500x<br><br>Better one?

2233<br>spear	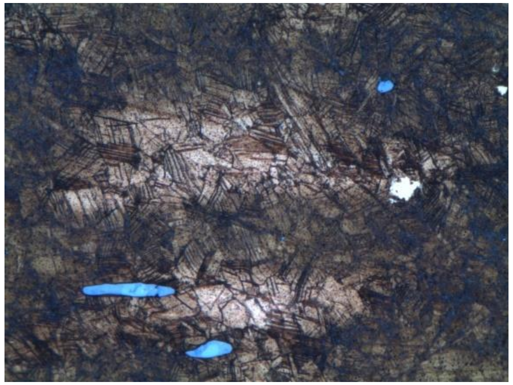	500x	2247<br>sickle	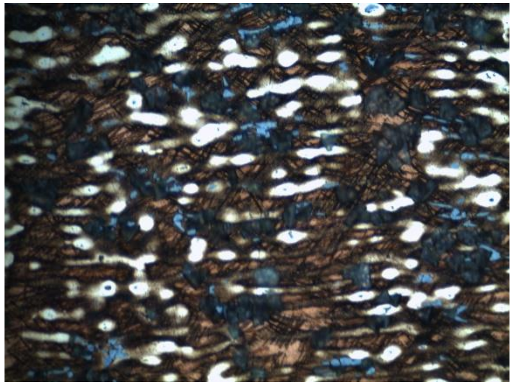	500x
2307<br>spear	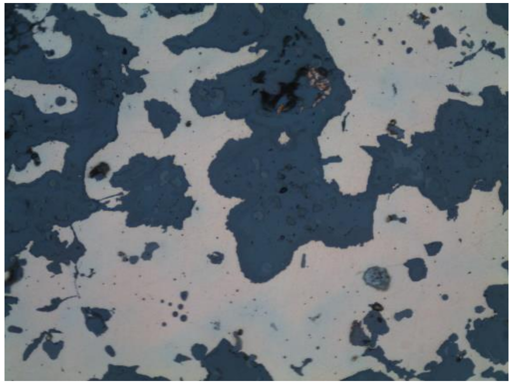	500x<br><br>Unetched micro-structure (more diagnostic than the etched structure)	2484<br>sickle	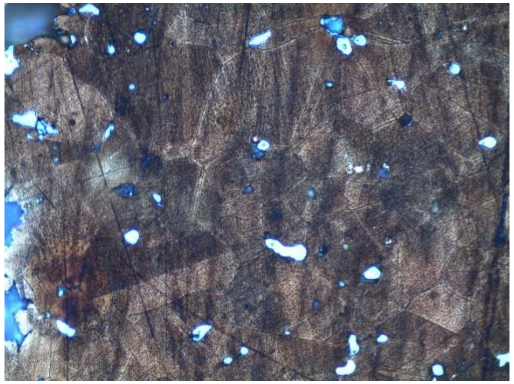	500x
2489<br>sword	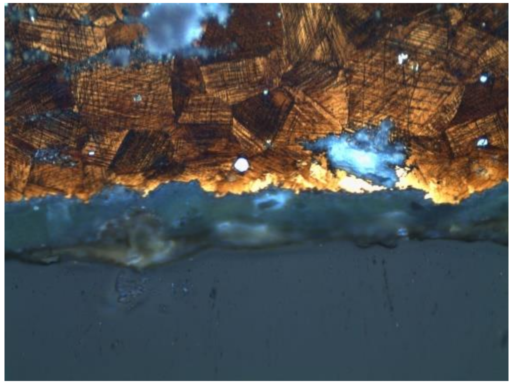	500x	2490<br>sword	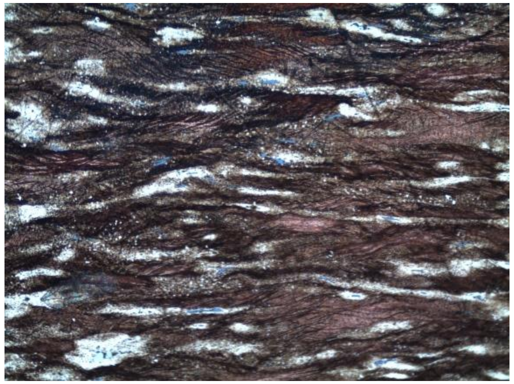	500x

27272 sword	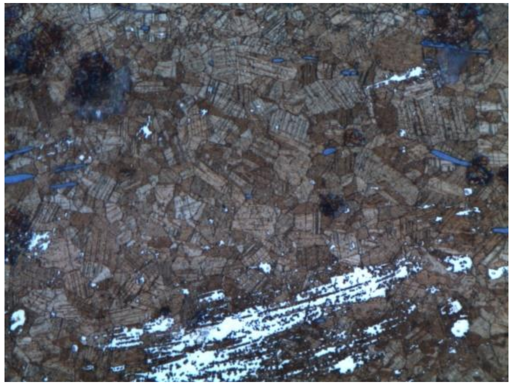	1000x	2883 sword	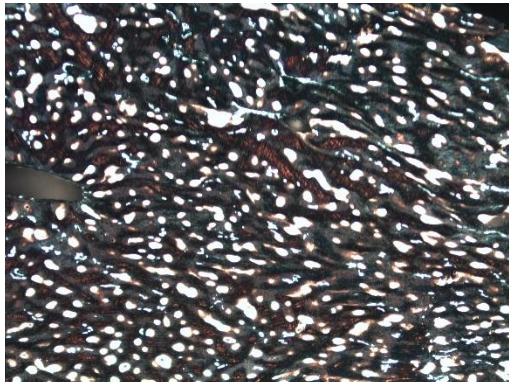	200x
2892 spear	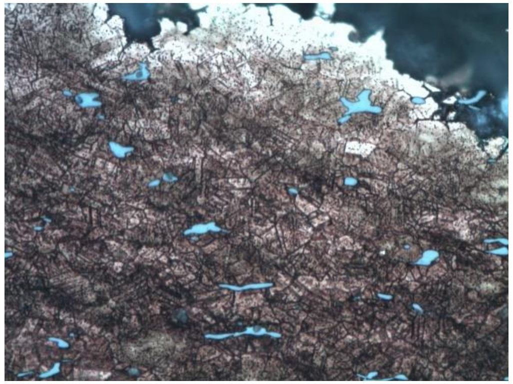	1000x	2896 spear	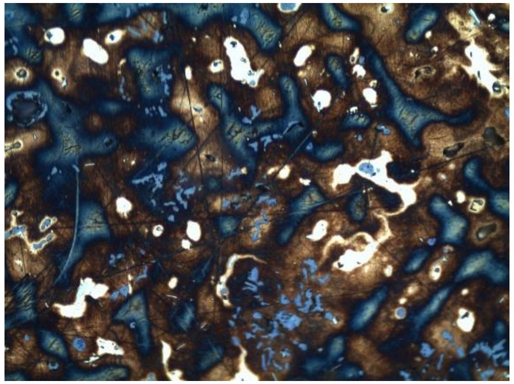	500x
2898 spear	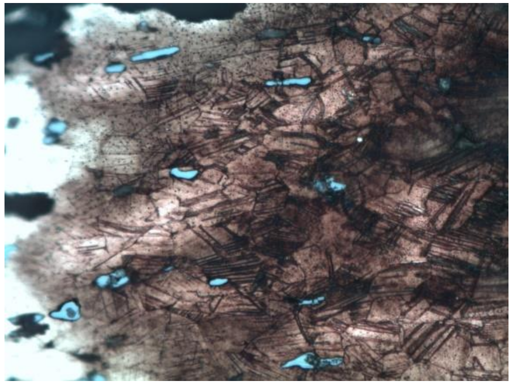	1000x	2926 axe	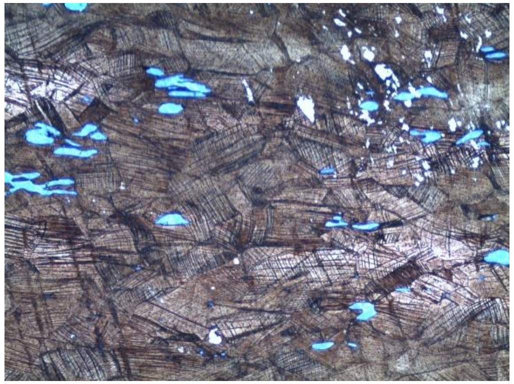	500x

2936<br>axe	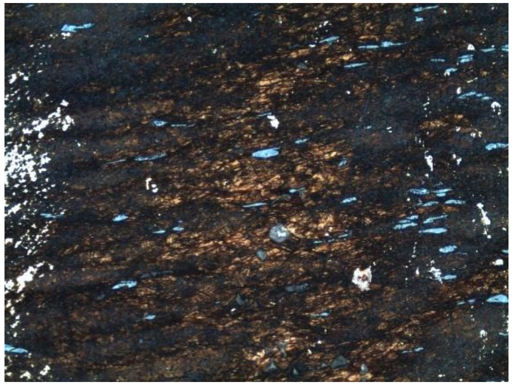	500x	3121<br>sword	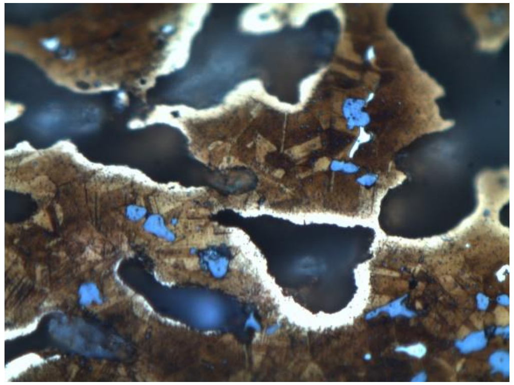	1000x
3222<br>spear	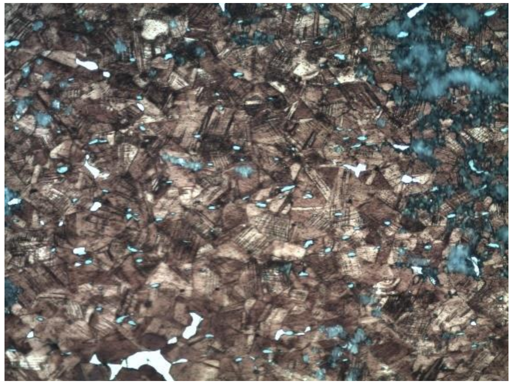	500x	3250<br>axe	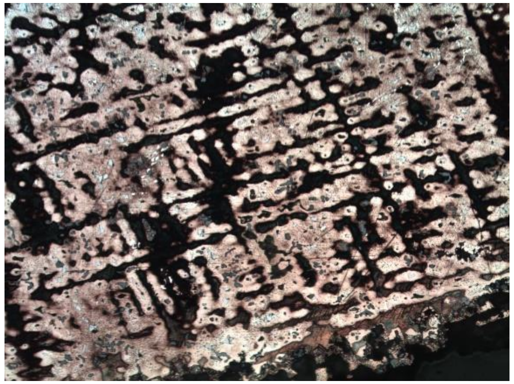	200x
3252<br>axe	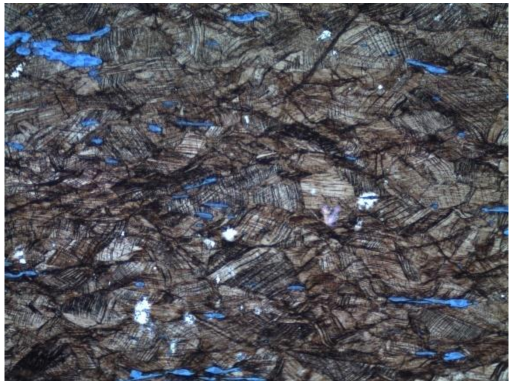	500x	3310<br>axe	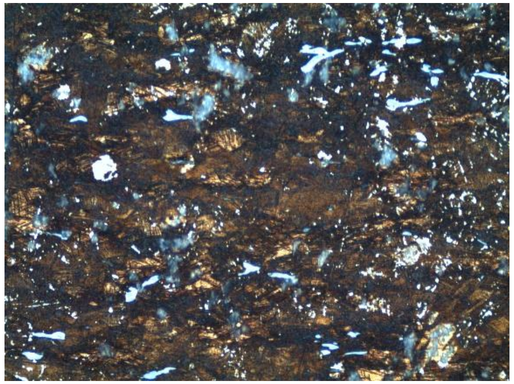	500x

3313<br>axe	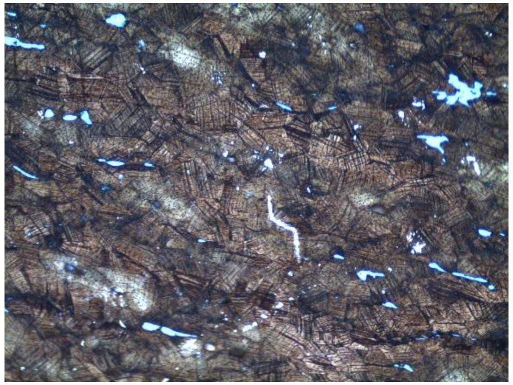	500x	3406<br>sword	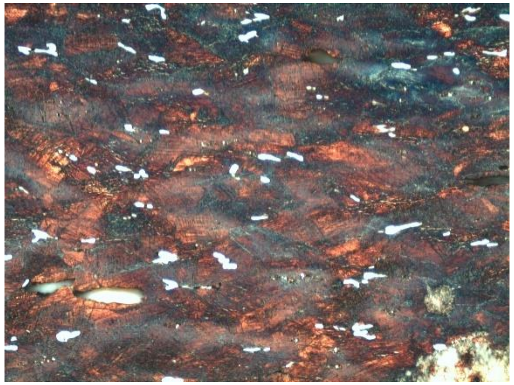	500x
3410-1<br>spear	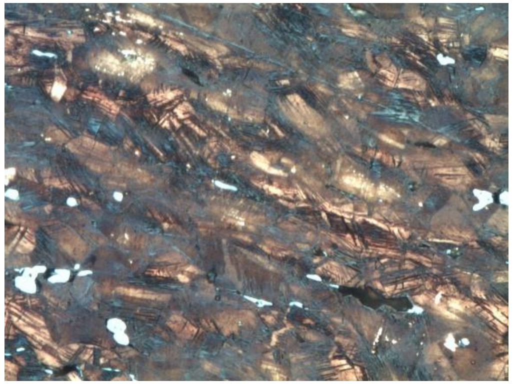	1000x	3412<br>spear	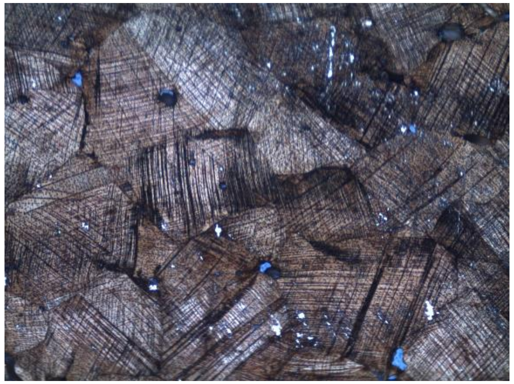	500x
3418<br>sickle	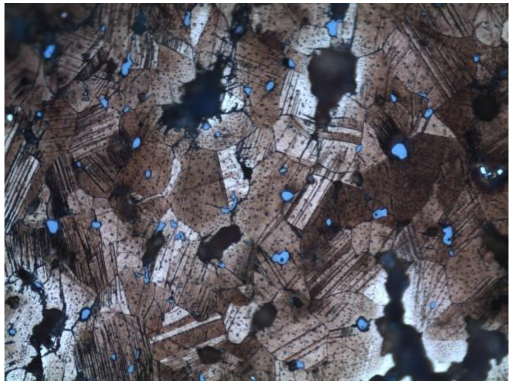	500x	3426<br>sickle	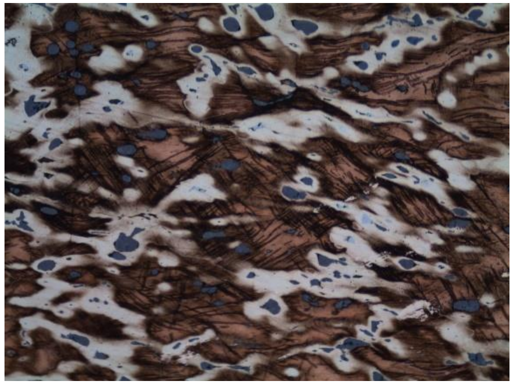	500x

3453 axe	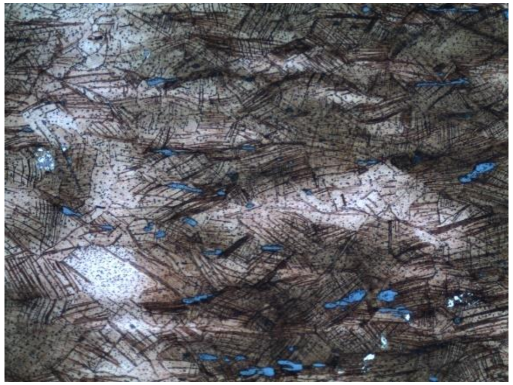	500x	3513 sword	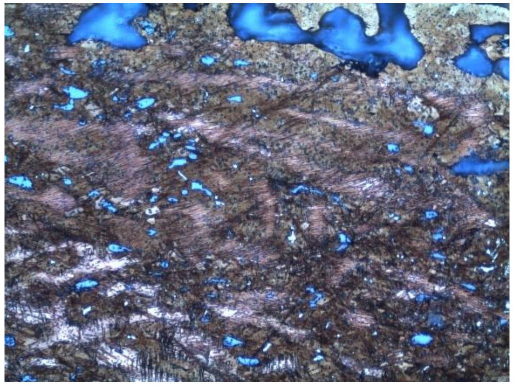	500x
3515 sword	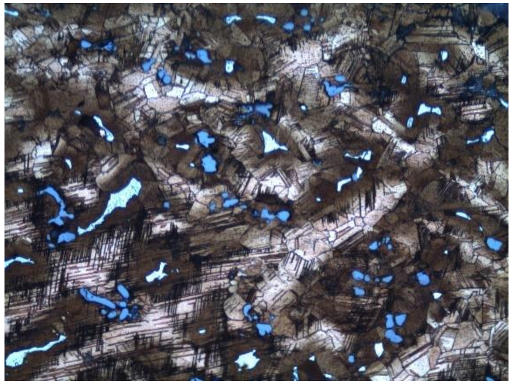	500x	3543 spear	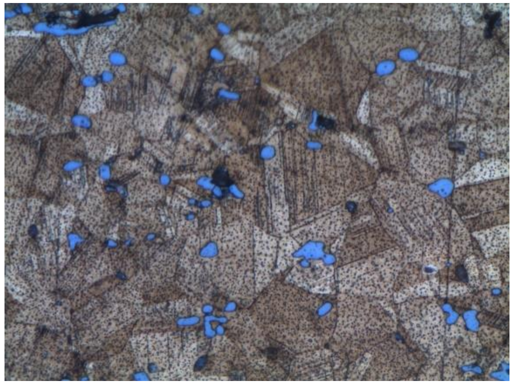	1000x
3548 spear	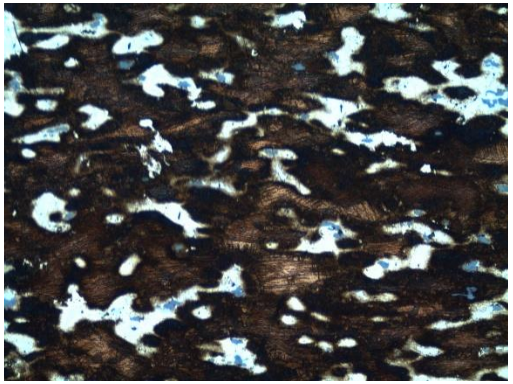	500x	3683 sword	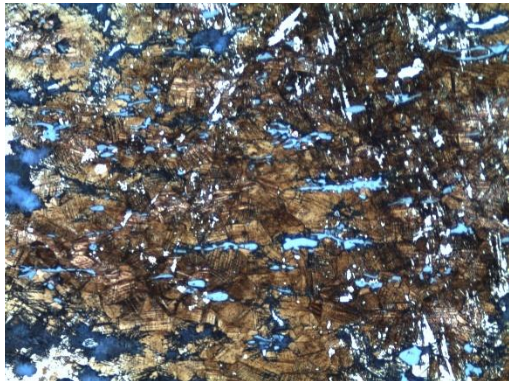	500x

3923<br>sword	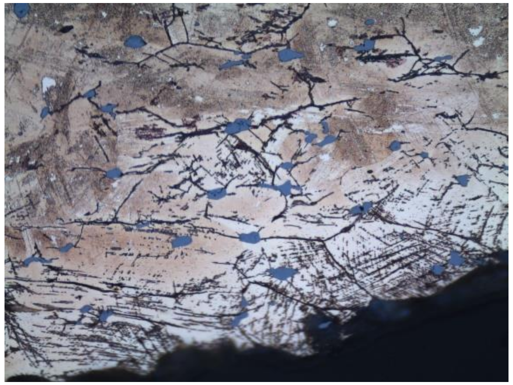	500x	3925<br>sword	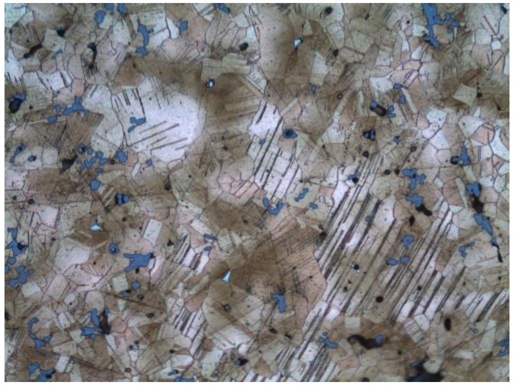	500x
3926<br>sword	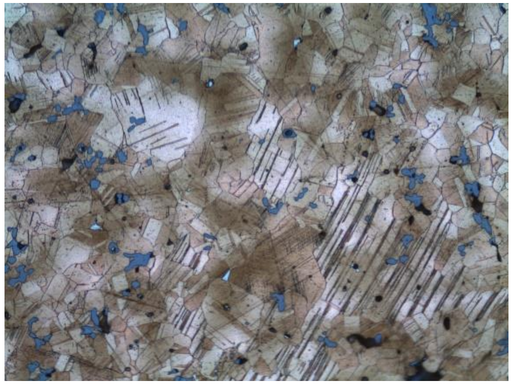	500x	3927<br>sword	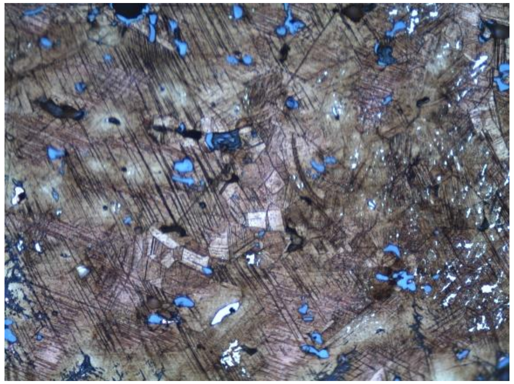	500x
3928<br>sword	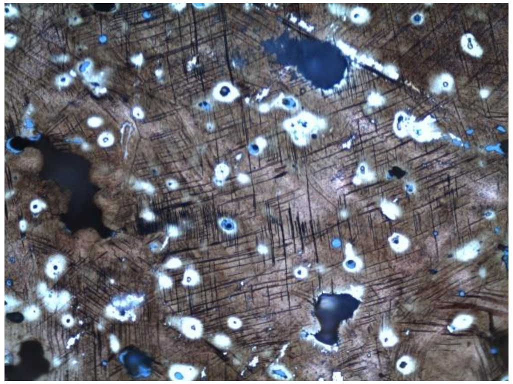	500x	3929<br>sword	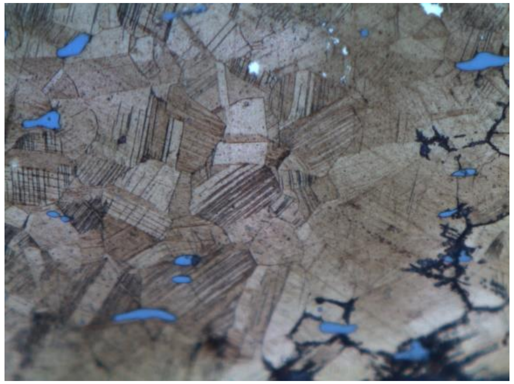	1000x

3939 spear	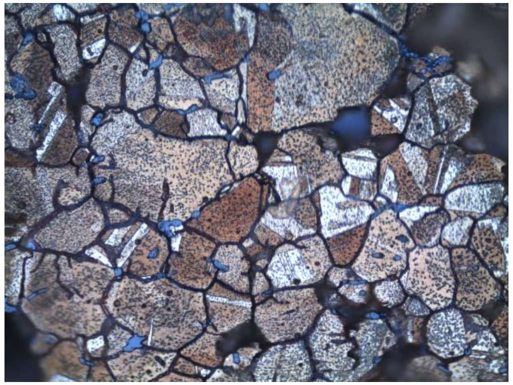	500x	3943 spear	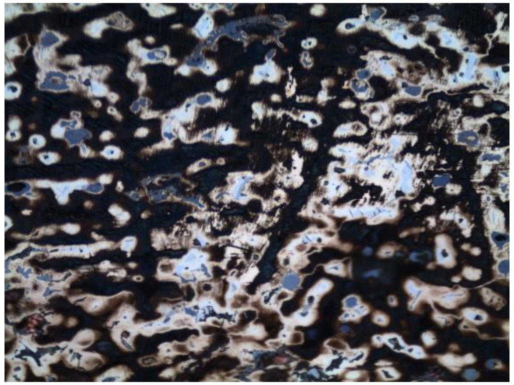	500x
3944 spear	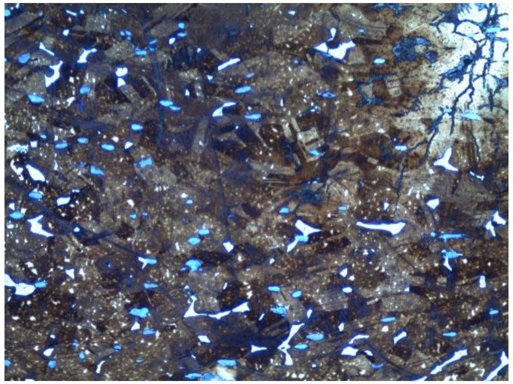	500x	3945 sword	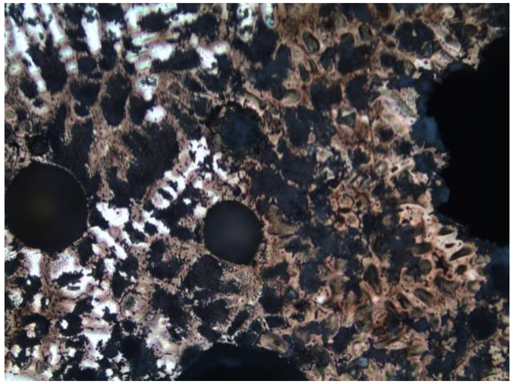	500x
3947 spear	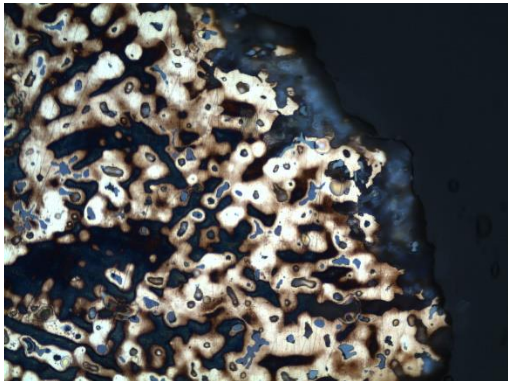	500x	4/725 spear	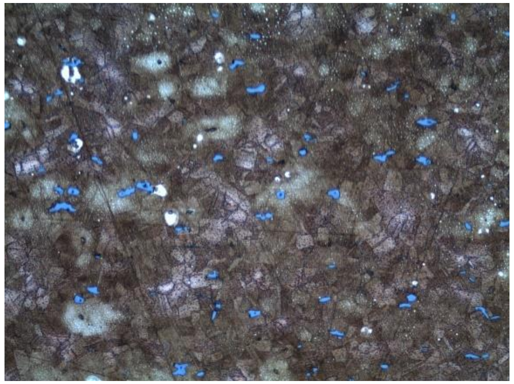	500x

45777<br>sword	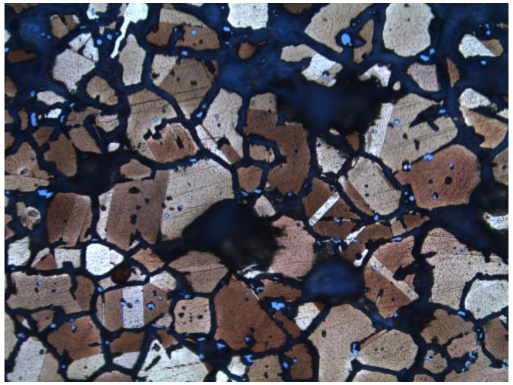	500x	6/153<br>spear	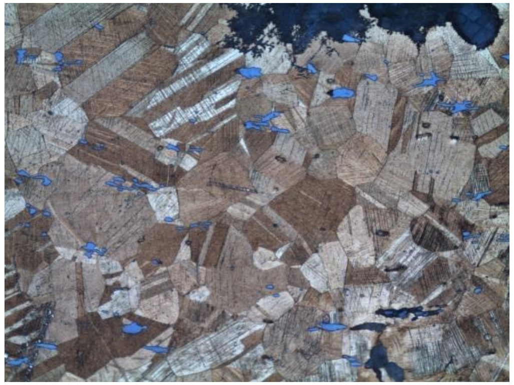	500x
6142<br>spear	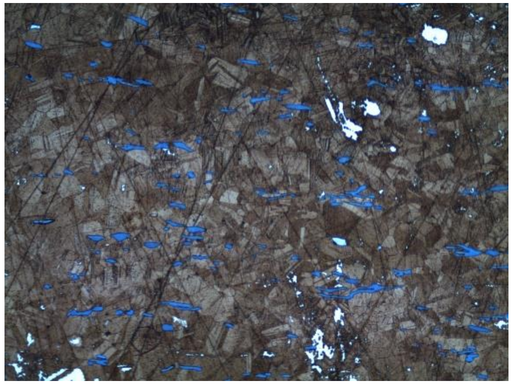	500x	6162<br>spear	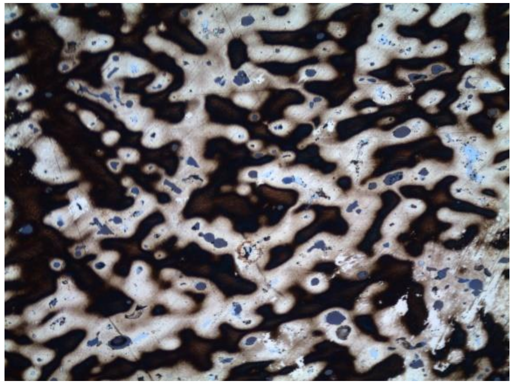	500x
6163<br>sword	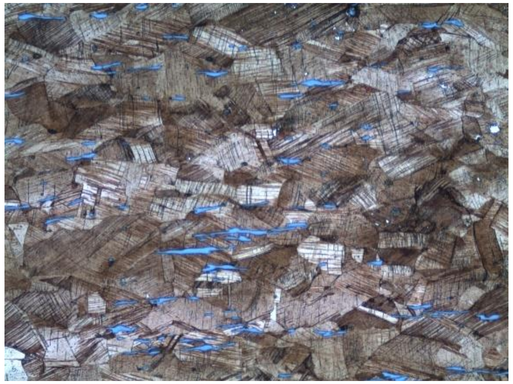	500x	6164<br>sword	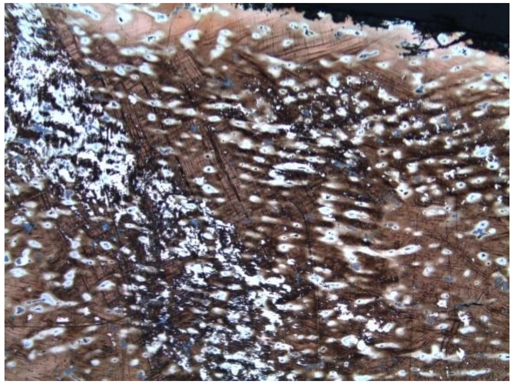	200x

700<br>sword	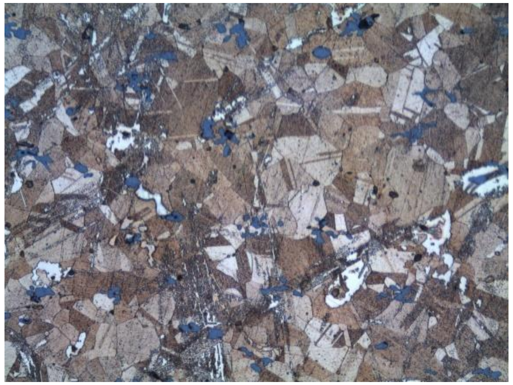	500x	8050<br>spear	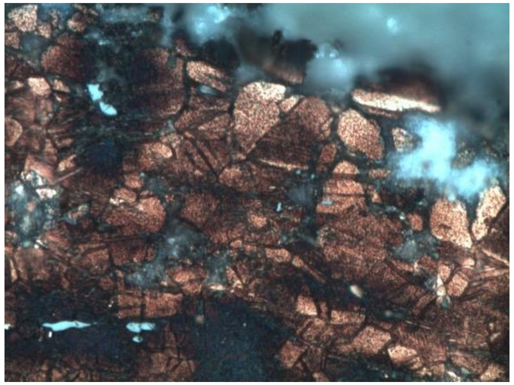	1000x
8051<br>sickle	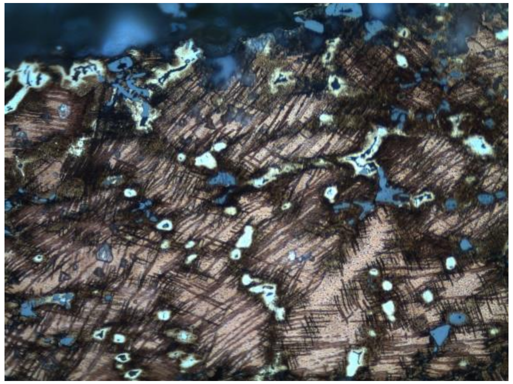	500x	8052<br>sickle	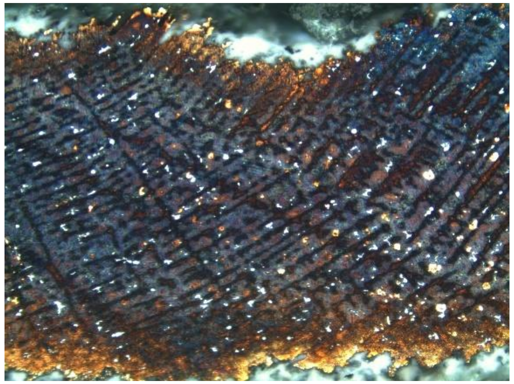	200x
869/AP1144<br>sickle	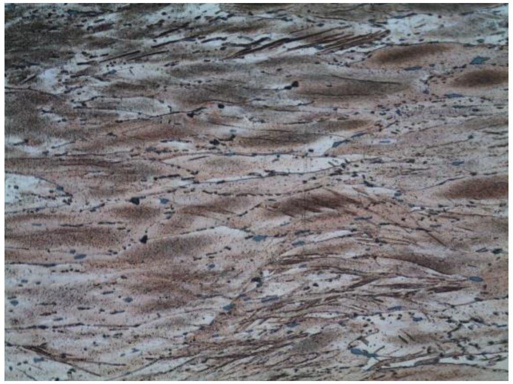	500x	876<br>spear	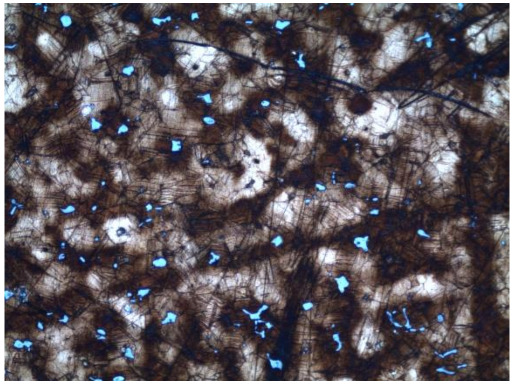	500x

893<br>spear	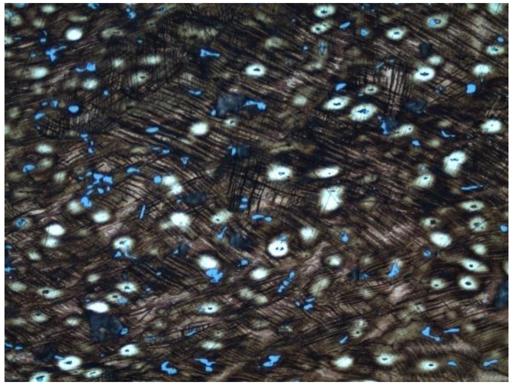	500x	896<br>axe	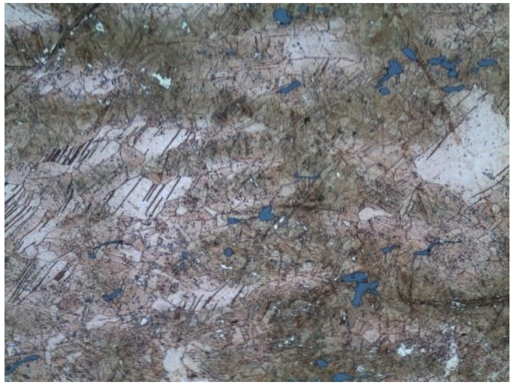	500x
A2969<br>sword	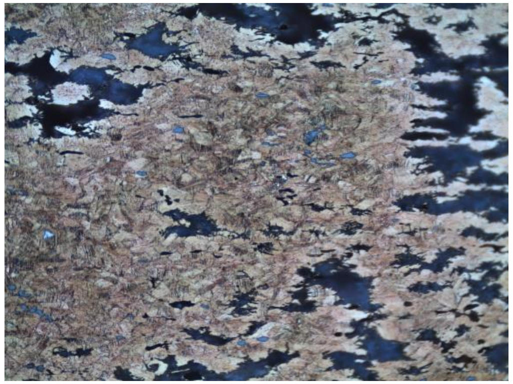	500x	A2994<br>sword	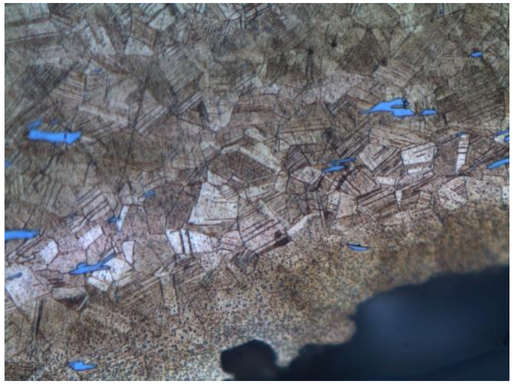	500x
A322<br>sword	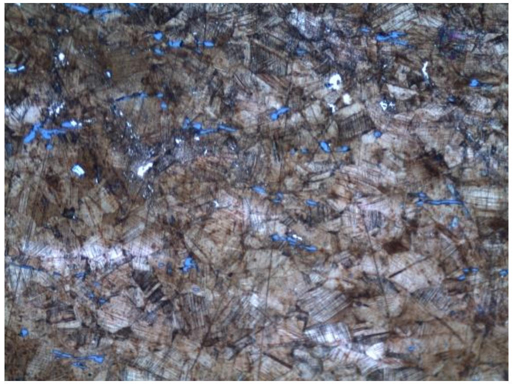	500x	A5282<br>spear	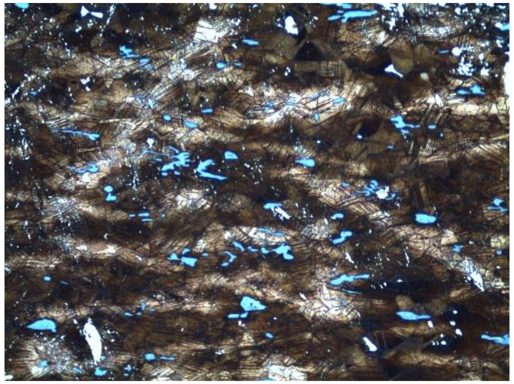	500x

AP10398 sickle	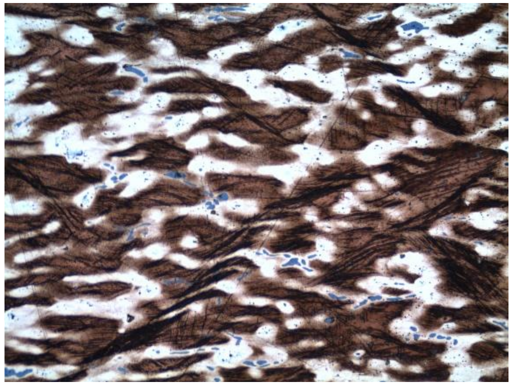	500x	AP10399 sickle	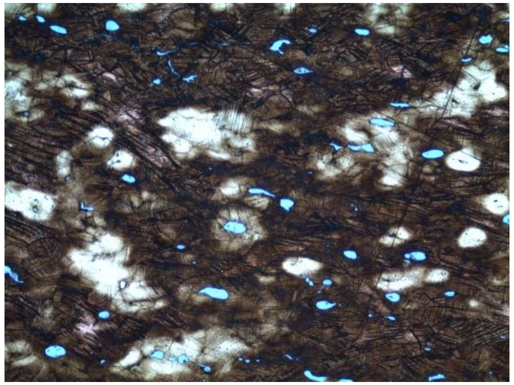	500x
AP10415 sickle	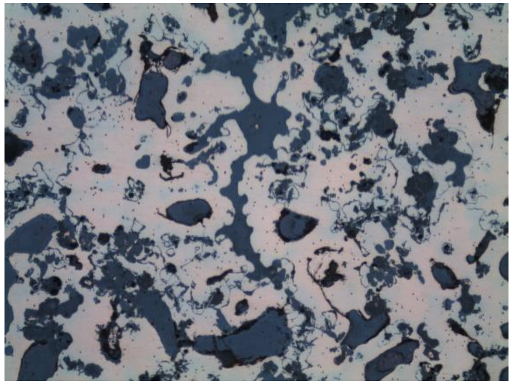	500x<br><br>Unetched structure included due to the high volume of inter-dendritic corrosion.	AP10416 sickle	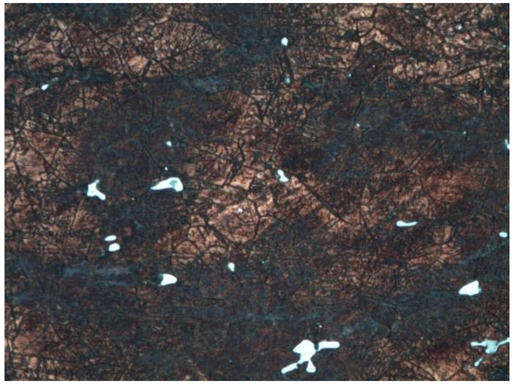	1000x
AP10473 sword	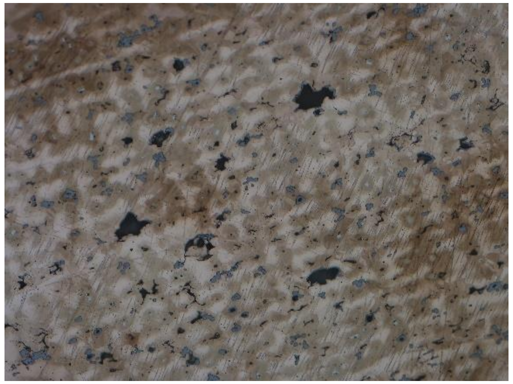	200x	AP10476 sword	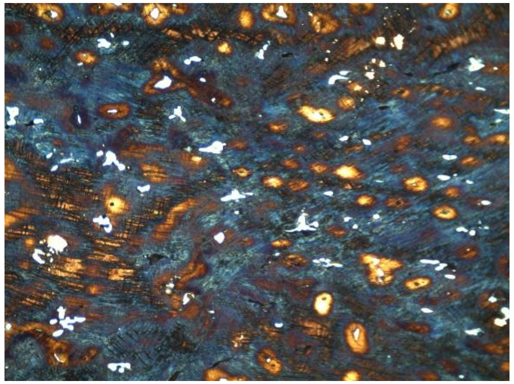	500x

AP10477 sword	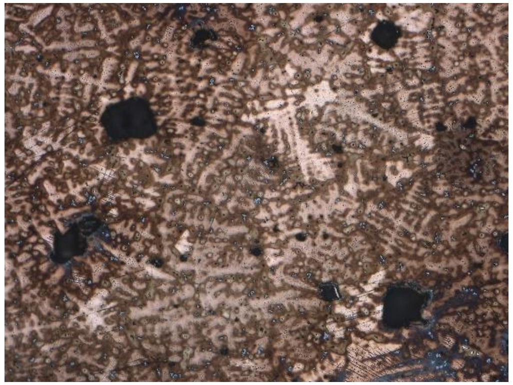	100x	AP10482 sword	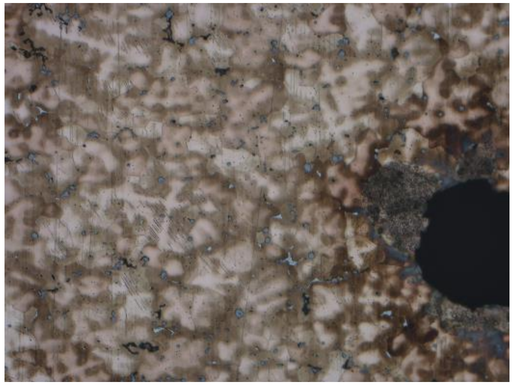	200x
AP10483 sword	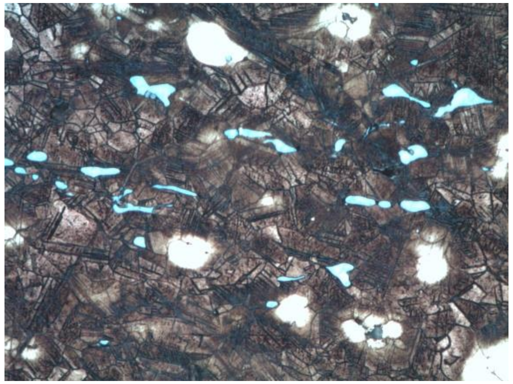	1000x	AP10614 spear	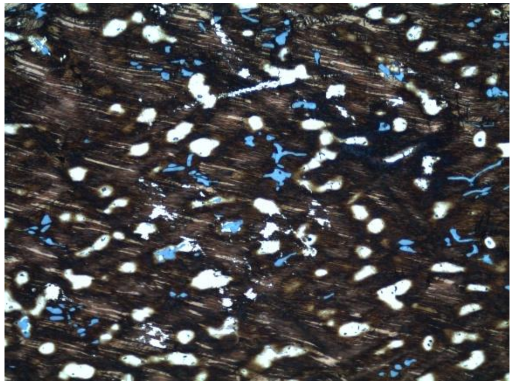	500x
AP10617 spear	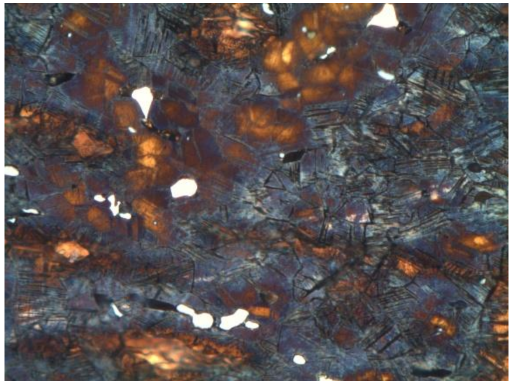	1000x	AP10618 spear	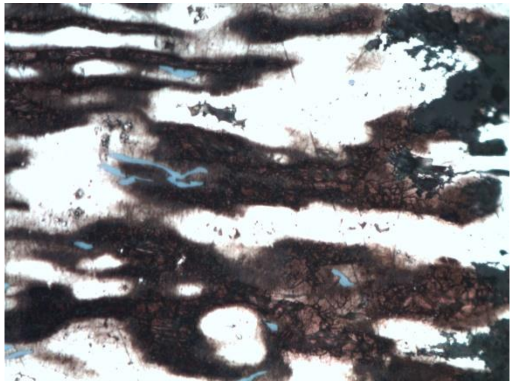	1000x

AP10623 spear	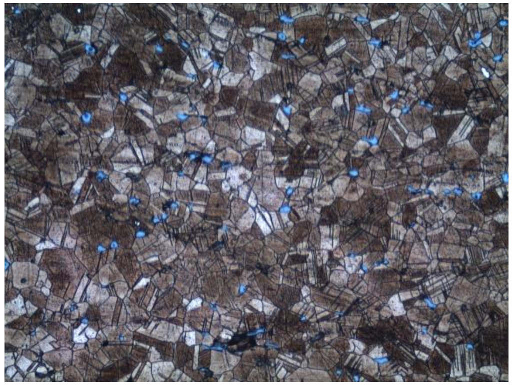	500x	AP10627 spear	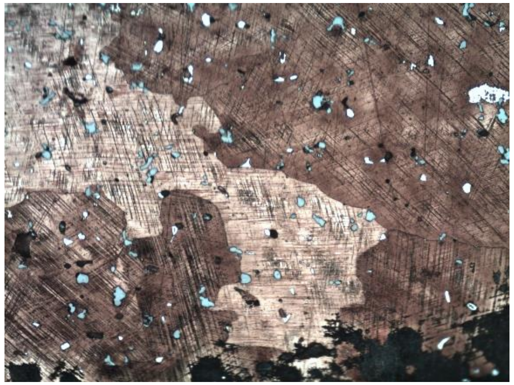	500x
AP10634 axe	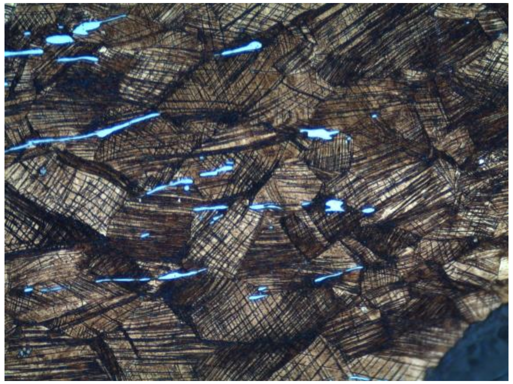	500x	AP10636 axe	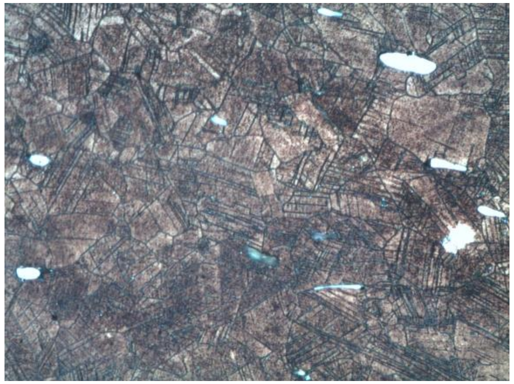	1000x
AP10638 axe	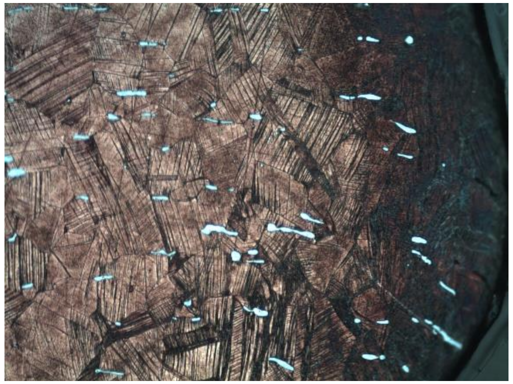	500x	AP10650 axe	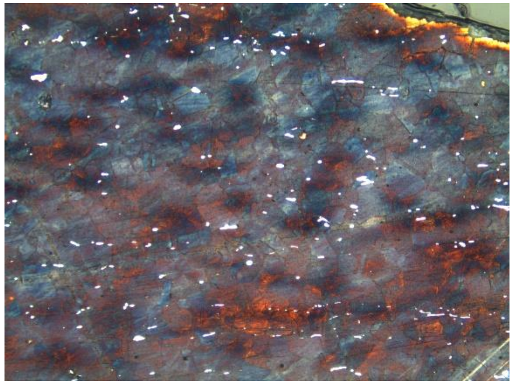	200x<br><br>Structure over-exposed to etchant.

AP1163<br>sword	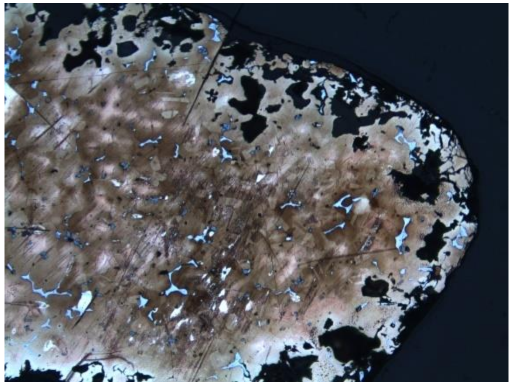	200x	AP1177<br>sword	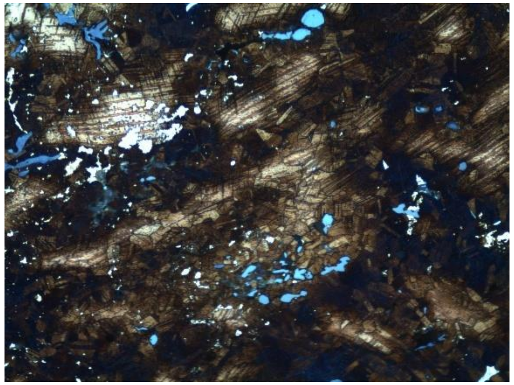	500x
AP1282<br>sickle	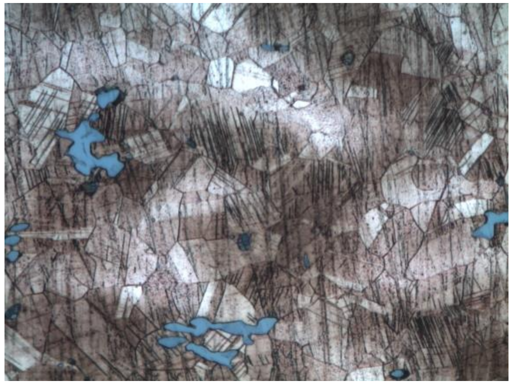	1000x	AP1283<br>sickle	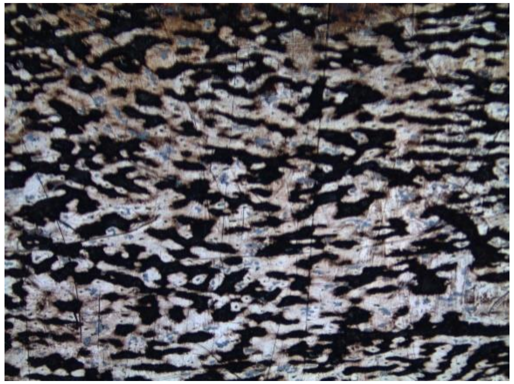	200x
AP1293<br>spear	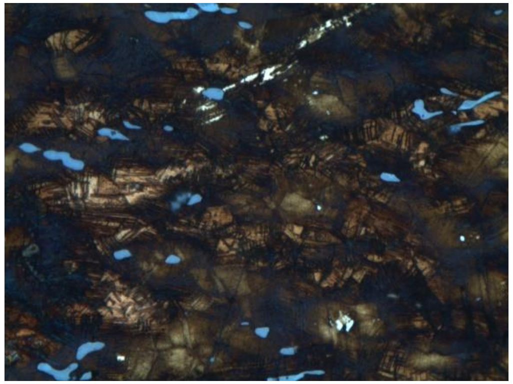	1000x	AP1296<br>spear	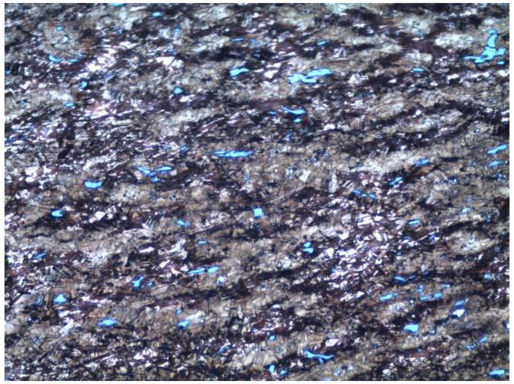	500x

AP1297 spear	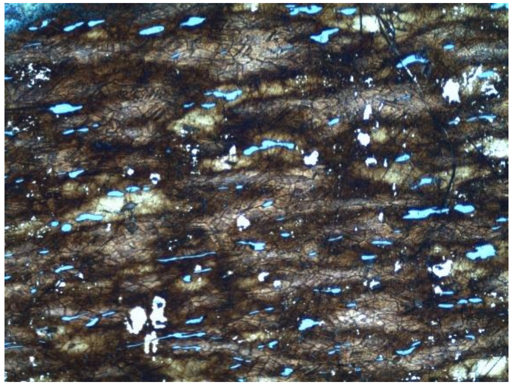	500x	AP1300 sword	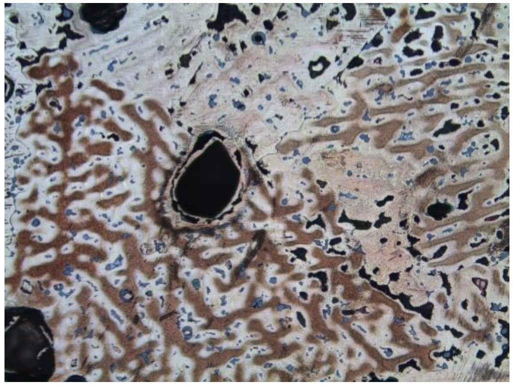	200x
AP3183 spear	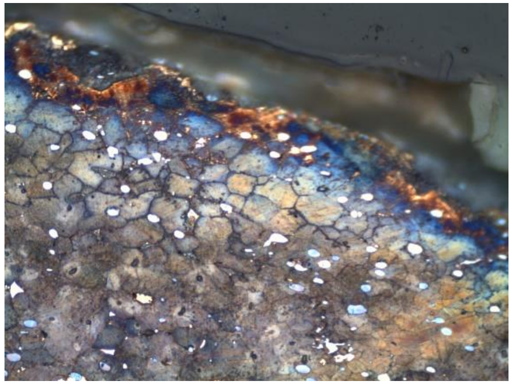	500x	AP3184 spear	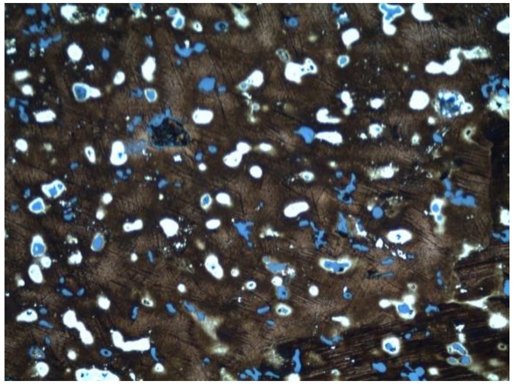	500x
AP3187 spear	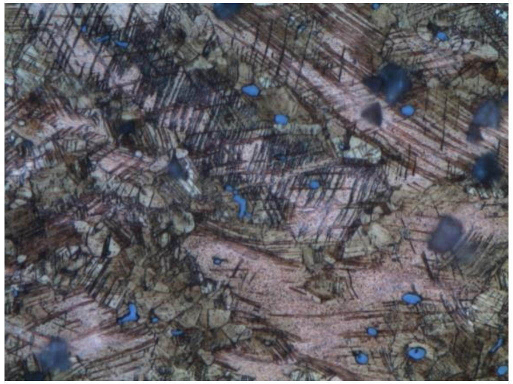	100x	AP3188 spear	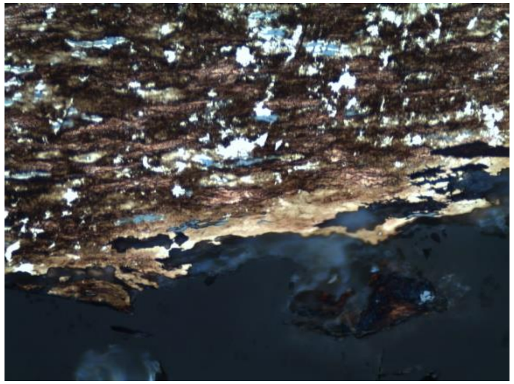	1000x

AP3189<br>spear	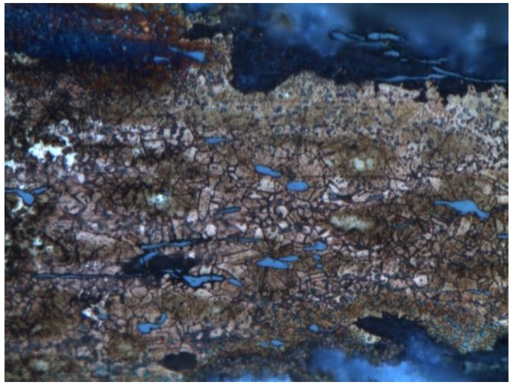	1000x	AP3190<br>spear	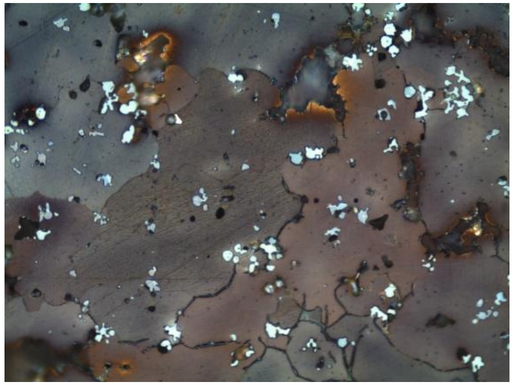	500x
AP3198<br>sword	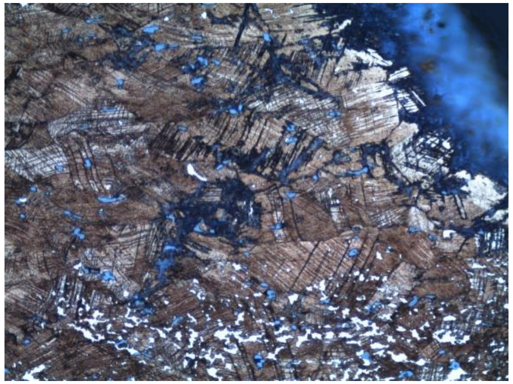	500x	AP3199<br>sword	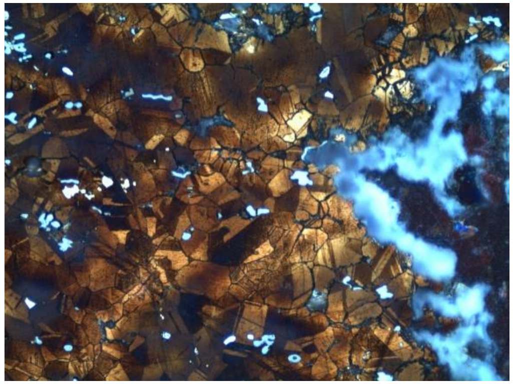	500x
AP3200<br>sword	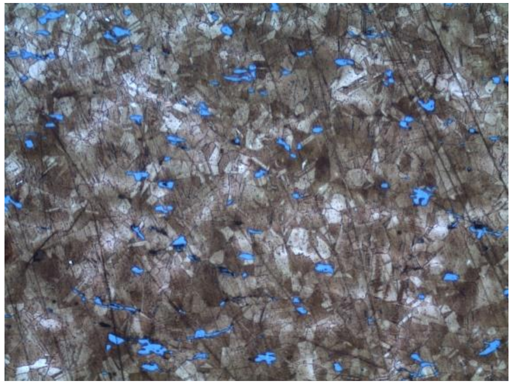	500x	AP3246<br>sword	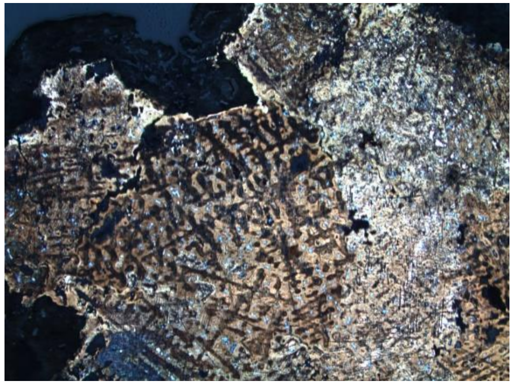	100x

AP3367 sword	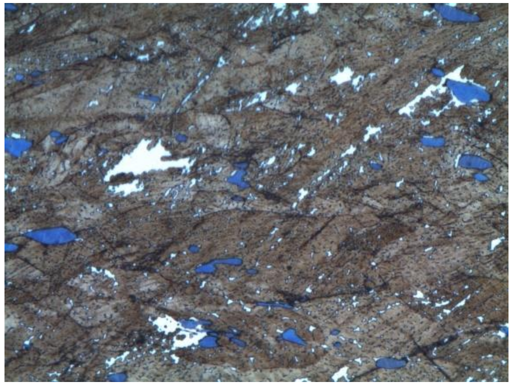	1000x	AP3369 sword	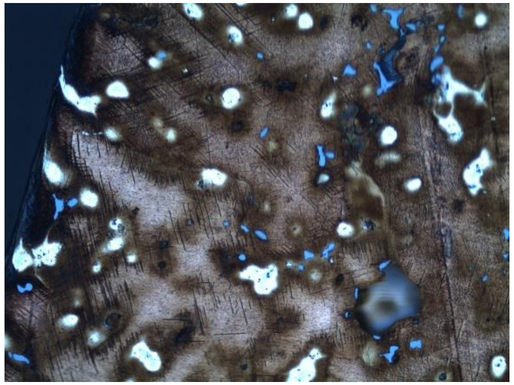	500x
AP3370 sword	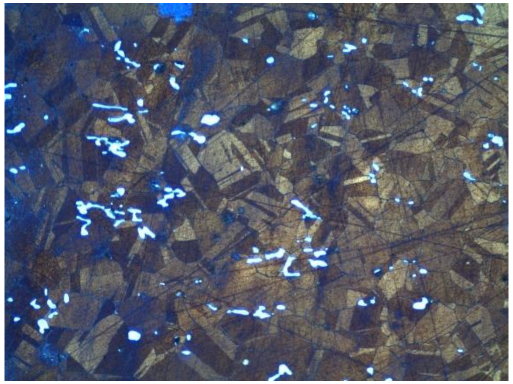	500x	AP3372 sword	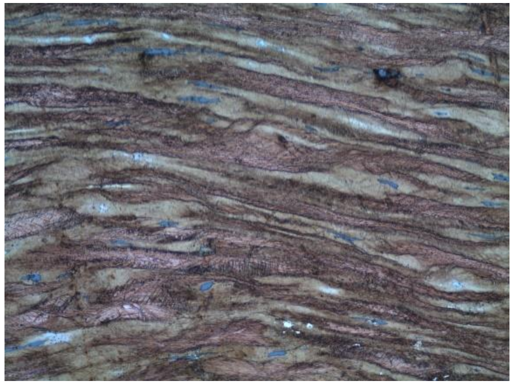	500x
AP3373 sword	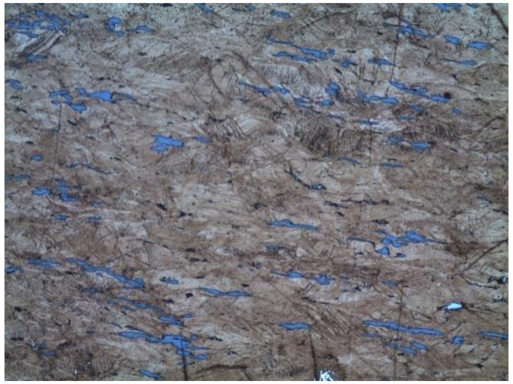	500x	IND JIJA sword	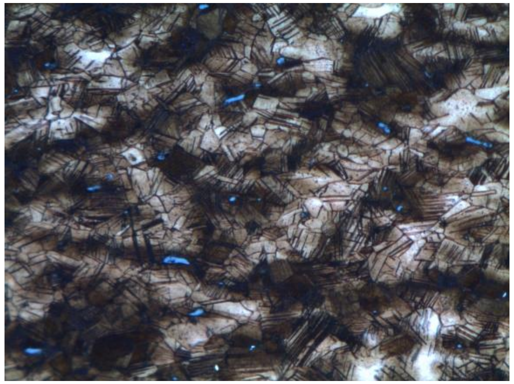	1000x

NN1<br>sickle	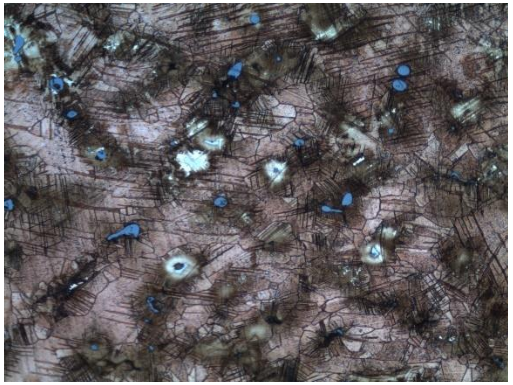	500x	NN2/1547.ii<br>axe	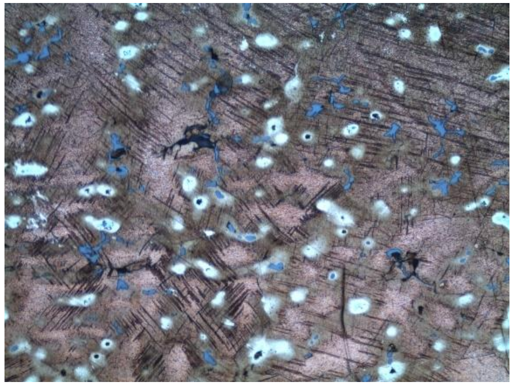	500x
P069<br>spear	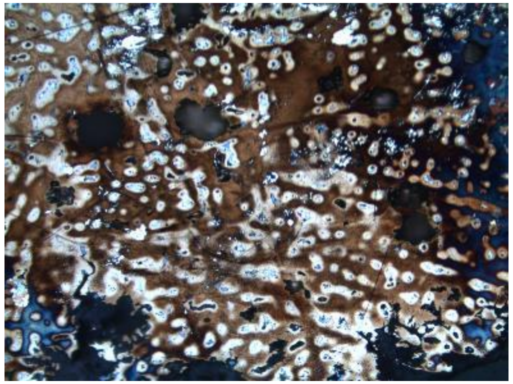	200x	P1034<br>sword	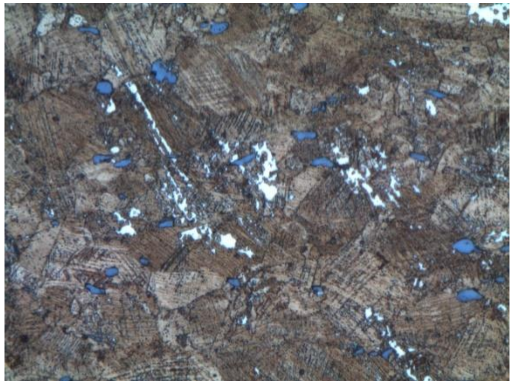	1000x
P1380<br>spear	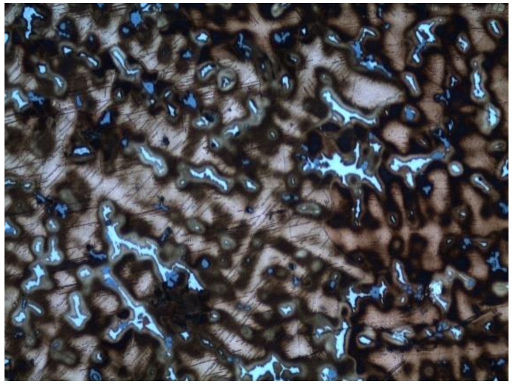	500x	P2639<br>spear	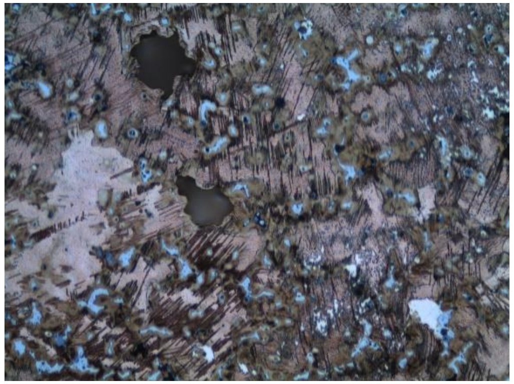	500x

P2640 sword	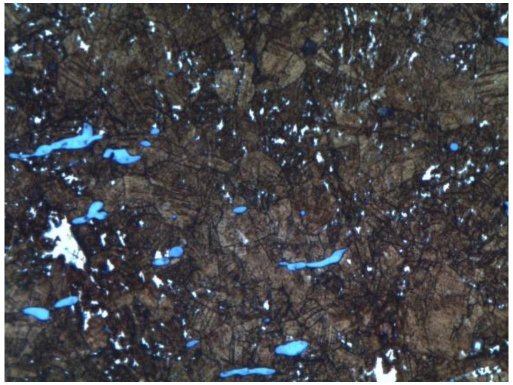	1000x	P341 sword	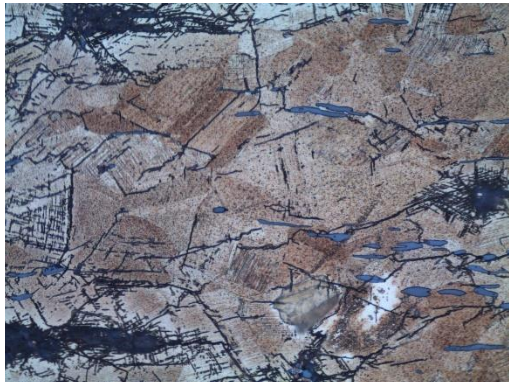	500x
P342 sword	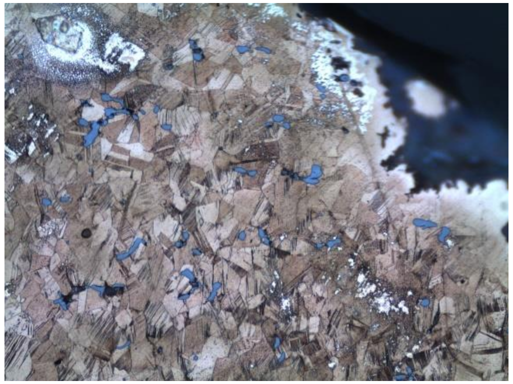	500x	P349 spear	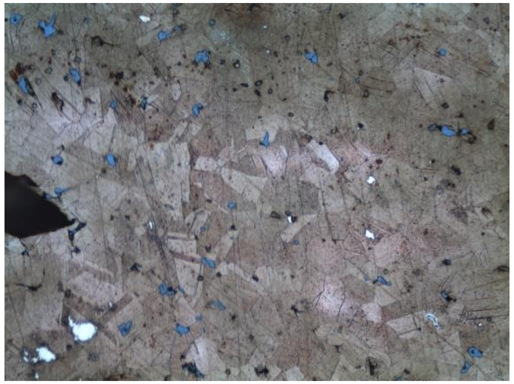	500x
P392 spear	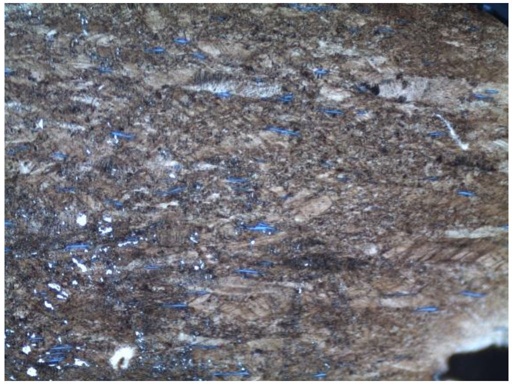	500x	P464 sword	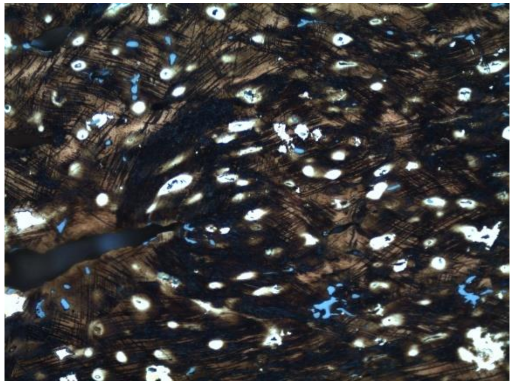	500x

P465 sword	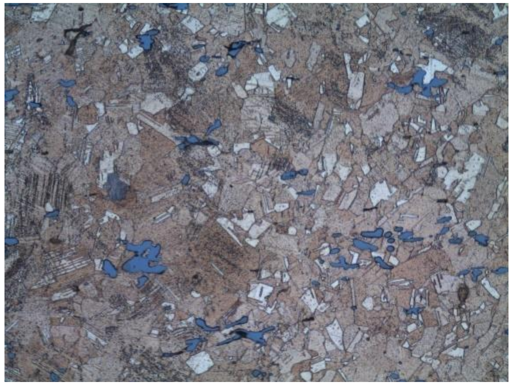	500x	P466 sword	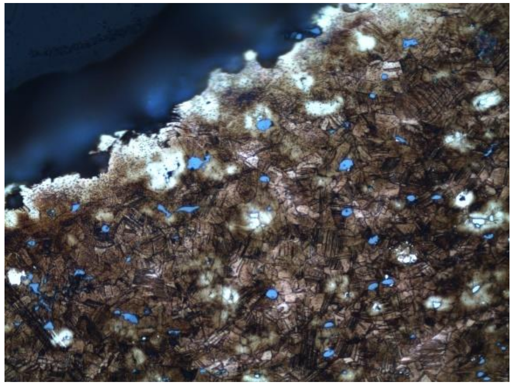	500x
P468 sword	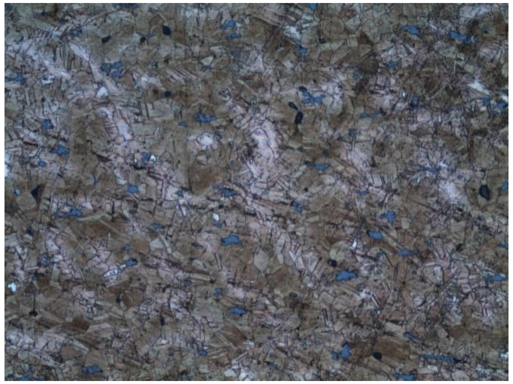	500x	P469 sword	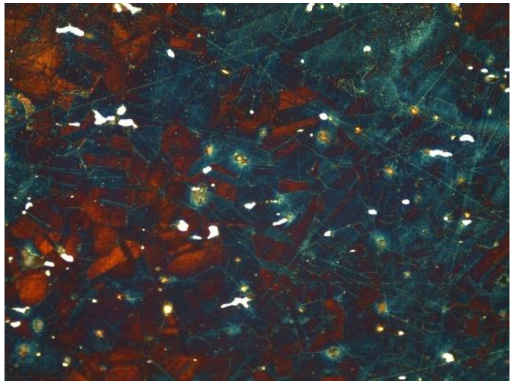	500x (over-etched)
P470 sword	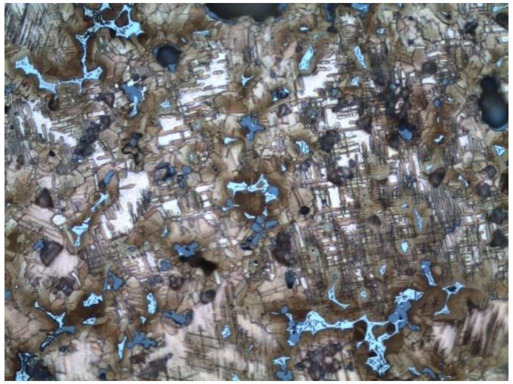	500x	P658 sword	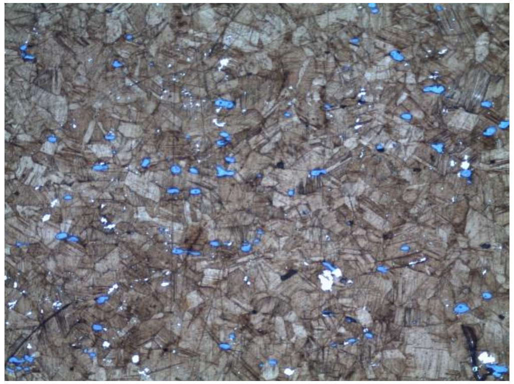	500x

P659 sword	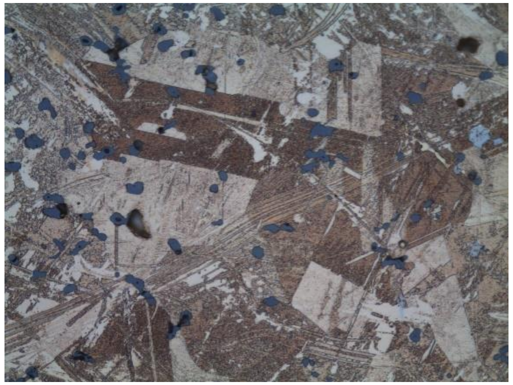	500x	P904 sword	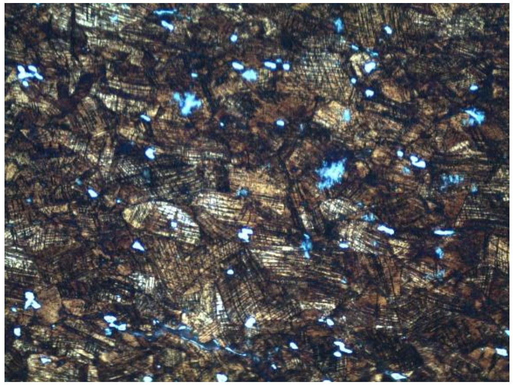	500x
P906 sword	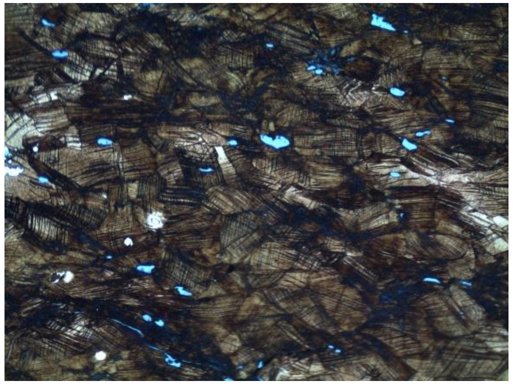	500x	P907 sword	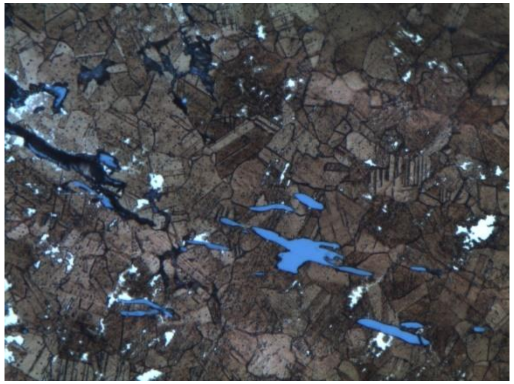	1000x
P908 1/2 spear	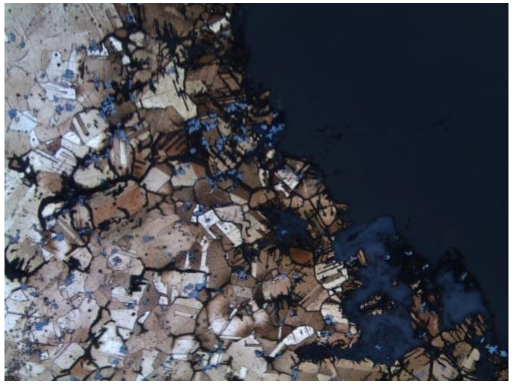	200x	P908 2/2 spear	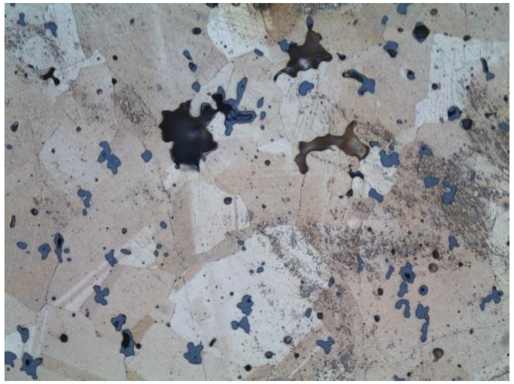	500x

SH2 spear	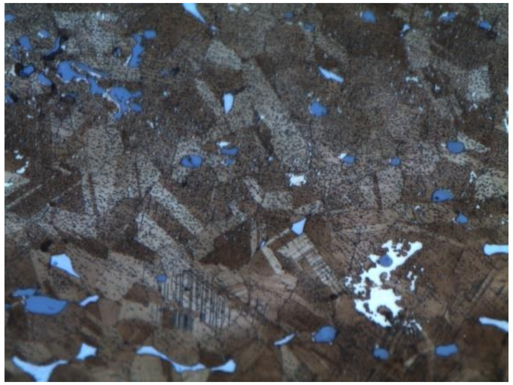	1000x	SH3 spear	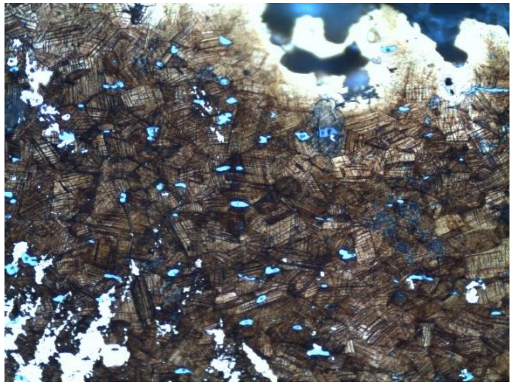	500x
SH4 spear	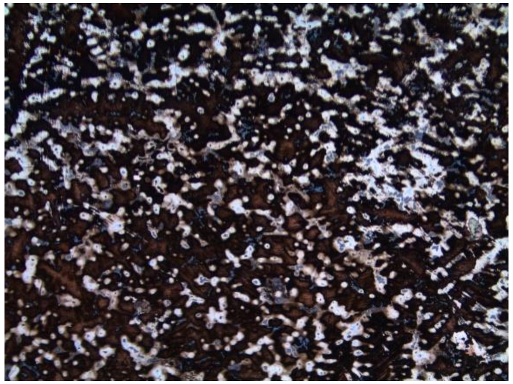	200x	UGR100 spear	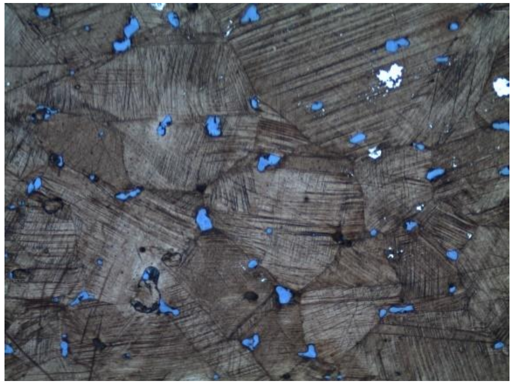	500x
UGR102 spear	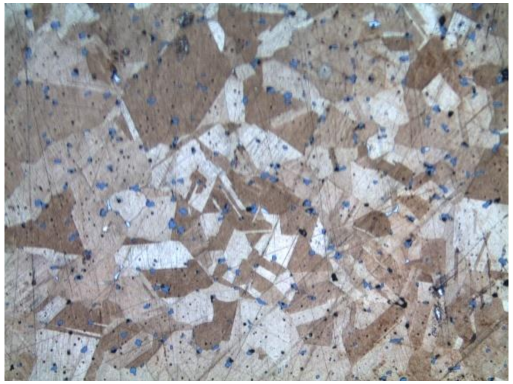	200x	UGR103 spear	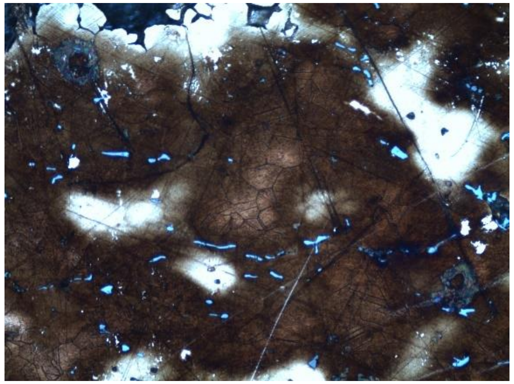	500x

UGR94 spear	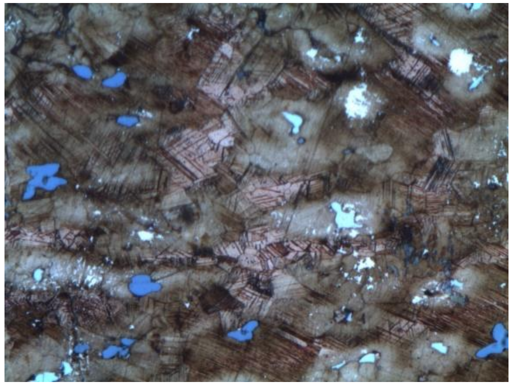	1000x	UGR95 spear	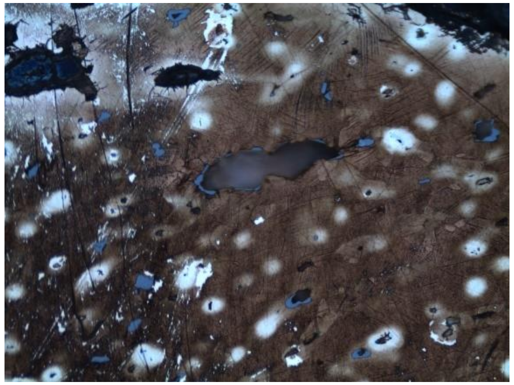	500x
